# A human intestinal epithelial-mesenchyme-immune triple culture system for disease modelling

**DOI:** 10.3389/fcell.2026.1867636

**Published:** 2026-07-07

**Authors:** Joep Korsten, Alessandro Dei, Carlemi Calitz, Nina Johannesson, Eline Freeze, Eloi Mercier, Jessica Chen, Mark Hills, Allen Eaves, Sharon Louis, Ryan K. Conder, Wing Chang, Dasja Pajkrt, Katja C. Wolthers, Salvatore Simmini, Adithya Sridhar

**Affiliations:** 1 OrganoVIR Labs, Emma Children’s Hospital, Department of Pediatric Infectious Diseases, Amsterdam UMC, Academic Medical Center, Amsterdam Institute for Infection and Immunity, Amsterdam Institute for Reproduction and Development, University of Amsterdam, Amsterdam, Netherlands; 2 OrganoVIR Labs, Department of Medical Microbiology, Amsterdam UMC, Academic Medical Center, Amsterdam Institute for Infection and Immunity, University of Amsterdam, Amsterdam, Netherlands; 3 STEMCELL Technologies UK Ltd., Cambridge, United Kingdom; 4 STEMCELL Technologies Inc., Vancouver, BC, Canada; 5 Terry Fox Laboratory, BC Cancer, Vancouver, BC, Canada; 6 Emma Center for Personalized Medicine, Amsterdam UMC, Amsterdam, Netherlands

**Keywords:** cytomegalovirus, dendritic cell, enterovirus, gut-on-chip, intestinal epithelial barrier, intestine-on-chip, macrophage, mesenchyme

## Abstract

**Introduction:**

The intestinal mucosa plays a vital role in nutrient absorption, drug metabolism, and pathogen defence. Advances in single-cell technologies have highlighted the specialised roles of various cell types that execute these diverse functions. Beyond intestinal epithelial cells, fibroblasts regulate the extracellular matrix (ECM) and modulate pro-inflammatory signalling, while antigen-presenting cells (macrophages and dendritic cells) maintain intestinal homeostasis and immune responses. However, the incorporation of such cellular complexity within existing *in vitro* models of the human intestine is currently lacking.

**Methods:**

We developed a human intestinal co-culture model that incrementally mimics the mucosal cellular environment, comprising intestinal epithelial cells, intestinal fibroblasts, and antigen-presenting cells. The model incorporated co-cultures of both adult and foetal cells to facilitate studies of intestinal development, barrier function, inflammation, and viral infections.

**Results:**

We successfully established an advanced multi-cellular intestinal co-culture model that recapitulates key features of the native mucosal environment. This model demonstrated the capacity for ECM deposition, Paneth cell differentiation, and functional immune interactions. Notably, the co-culture system effectively modelled immune responses during viral infections, demonstrating the utility of incorporating multiple cell types and developmental stages.

**Discussion:**

Our co-culture model improves the physiological relevance of in vitro studies by incorporating multi-cellular complexity previously lacking in existing systems. This advancement enables the exploration of epithelial-mesenchymal-immune crosstalk in intestinal health and disease, providing a powerful platform for studying intestinal development, barrier dysfunction, and infection-related pathology.

## 1 Introduction

The mucosal lining of the human gastrointestinal (GI) tract is the largest tissue exposed to the external environment. It plays a crucial role in nutrient absorption and the first-pass metabolism of drugs while protecting non-mucosal sites from invading pathogens (Hickey et al., 2023; Antfolk and Jensen, 2020). The development and maintenance of the GI tract depend not only on the intestinal epithelial cell (IEC) lining but also on intricate interactions between IECs and cells from other germ layers. Intestinal fibroblasts (IFs) form and manage the dynamic extracellular matrix (ECM) of the intestinal mucosa, providing essential structural support to the IECs (Zhu et al., 2021). Additionally, IFs secrete immunomodulatory cytokines, which are fundamental not only during development to preserve immune cell tolerance, but also in the establishment of inflammatory response during injury and infections (Rees et al., 2020). The intestinal mucosa is the largest immune organ and is a reservoir for 75% of the immune cells in the body. In particular, antigen-presenting cells (APCs), such as macrophages (MΦs) and dendritic cells (DCs) play critical roles in mucosal immunity. The phenotypical and functional plasticity of these cells is central to maintaining intestinal homeostasis and responding to environmental challenges (Smythies et al., 2005; Filardy et al., 2023). Under steady-state conditions, MΦs maintain intestinal health by recognising and eliminating pathogens that breach the intestinal barrier, clearing apoptotic cells and debris, enabling tolerance to commensal microbiota and food antigens, and preventing unnecessary inflammation through the production of anti-inflammatory cytokines, like interleukin 10 (IL-10) (Shouval et al., 2014). Under similar homeostatic conditions, DCs, specialised in sampling luminal antigens using pattern recognition receptors (PRRs), maintain a tolerogenic phenotype that facilitates immune tolerance and induction of regulatory T cells (Rua et al., 2011). However, following tissue damage, infections, autoimmune reactions, and certain environmental factors this tolerogenic environment can be disrupted, leading to the activation of certain PRR-associated pathways, ensuing antigen processing and presentation in Peyer’s patches or mesenteric lymph nodes. During this phase, APCs stimulate the recruitment and activation of effector T cells, promoting robust immune responses to pathogens or harmful antigens in the intestine (Gaudino and Kumar, 2019).

Human *in vitro* models that include this complex multicellular diversity and interactions are needed for a better understanding of intestinal development and disease states, including inflammation and infection (Liu et al., 2024). Although reductionist, *in vitro* models enable the study of complex molecular mechanisms and high-throughput drug development (Wu et al., 2023a). Currently, stem cell-derived human intestinal enteroids and organoids are emerging to be the gold standard *in vitro* models of the human intestine (Wang et al., 2023; Zou et al., 2017). However, these organoids do not capture the cellular complexity of the intestinal mucosa, as they primarily contain IECs (Taelman et al., 2022). A significant concern with these IEC-three-dimensional (3D) models is their short lifespan upon differentiation, ranging from less than 72 h to 7 days, due to rapid terminal differentiation of the IECs (Hickey et al., 2023). Furthermore, these 3D models have a closed cystic orientation that limits their utility for applications that focus on apical epithelial interactions. To circumvent these challenges, we and others have developed 2D human intestinal organotypic models on cell culture inserts that allow IEC polarisation and luminal accessibility (Roodsant et al., 2020; Stroulios et al., 2021). More recently, organ-on-chip (OoC) systems that partially recapitulate important features of the intestinal mucosa have also been developed. These intestinal OoC mouse models have been implemented to investigate how the interplay between IECs and IFs influences the cellular composition of the intestinal epithelium and to model tumour growth and invasion *in vitro* (Verhulsel et al., 2021). However, several barriers such as high costs, lack of standardisation, and a steep learning curve limit the widespread use of these OoC systems (Weindl et al., 2021; Akhtar et al., 2017).

**FIGURE 1:**
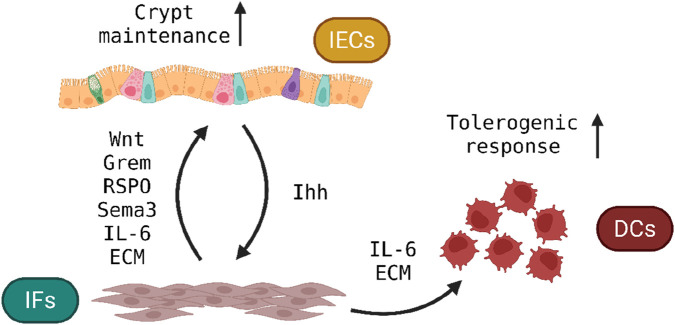
Schematic overview of the presumed interactions between the cell populations of the intestinal mucosa model (Pasztoi and Ohnmacht, 2022). Figure created using Biorender.

To address these limitations, we developed a human intestinal model that recapitulates the cellular complexity of the intestinal mucosa in a readily accessible and easy-to-use cell culture insert system. Specifically, we optimised and established co-culture models of adult and foetal cells to mimic IEC-IF, IEC-APC, and IEC-IF-APC interactions *in vitro*. In these models, IFs are expected to modulate IECs through the secretion of extracellular matrix (ECM) proteins, Wnt signalling activators, including R-spondins (Rspo1 & Rspo3) and class 3 semaphorins (Sema3), as well as BMP inhibitory signals such as Gremlin (Grem1 & Grem3), thereby aiding in the maintenance of intestinal crypts and culture longevity. Reciprocally, epithelial Indian hedgehog signalling (Ihh) is anticipated to promote IF proliferation. Beyond their structural support role, IFs are also expected to promote tolerogenic responses in APCs, shaping resident DC phenotypes (Pasztoi and Ohnmacht, 2022), creating a dynamic interplay between the different cell populations (Figure 1). Additionally, DCs are described as transferring viral particles to other cell types without undergoing viral replication themselves (Helgers et al., 2022). This capacity would make them an important component of an enhanced physiological intestinal infection model. The co-culture models in this study were thoroughly characterised, and their utility demonstrated for various applications, including studies on intestinal barrier development and function, inflammation, and viral infections.

## 2 Results

### 2.1 Epithelial-mesenchymal crosstalk regulates ECM organisation and intestinal barrier homeostasis

**FIGURE 2:**
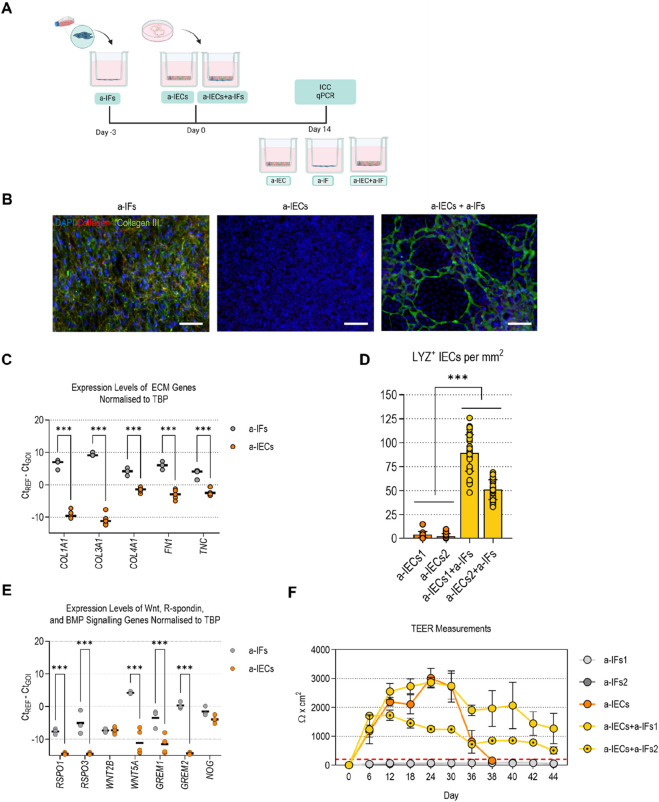
The epithelial-mesenchymal crosstalk regulates ECM organisation and intestinal barrier homeostasis in adult IEC-IF cultures. **(A)** Schematic representation of the workflow to establish a-IEC and a-IF co-cultures, along with their respective monoculture controls. **(B)** Representative immunofluorescence images of collagen type I and collagen type III deposition at day 14 in a-IFs, a-IECs, and a-IECs+a-IFs maintained in IMM (scale bars = 100 µm). **(C)** RT-qPCR analysis of ECM genes performed on a-IECs and a-IF monocultures maintained for 14 days on cell culture inserts in IMM. Gene expression levels are plotted as -ΔCt values. Each point represents a biological replicate (n = 3), orange and grey colours highlight data related to a-IFs and a-IECs, respectively. Gene names are listed in the x-axis. Statistical analysis employed a two-way ANOVA, along with Šídák’s multiple comparison test. **(D)** Bar graph summarising the results of the Paneth cell quantification based on LYZ immunostaining (biological n = 2). The bars represent the mean and standard deviation. Adult IECs derived from different donors are indicated with distinct numerical suffixes (e.g. a-IECs1 and a-IECs2). Statistical analysis employed a one-way ANOVA and Tukey’s multiple-comparison test. **(E)** RT-qPCR analysis of Wnt, R-spondin, and BMP signalling genes performed on a-IECs and a-IFs cultures maintained for 14 days on cell culture inserts in IMM. Gene expression levels are plotted as -ΔCt values. Each point represents a biological replicate (n = 3), orange and grey colours highlight data related to a-IFs and a-IECs, respectively. Gene names are listed in the x-axis. Statistical analysis employed a two-way ANOVA, along with Šídák’s multiple comparison test. **(F)** TEER measurements performed on a-IFs, a-IECs, and a-IECs+a-IFs maintained in the same medium. TEER measurements were normalised to the surface area of the cell culture inserts (0.332 cm^2^) and plotted as Ω·cm^2^ (y-axis) over time (x-axis). Each point represents the mean of at two technical replicates performed on different inserts, and the bars indicate the standard deviation of these measurements. Grey, orange, and yellow colours represent data for a-IFs, a-IECs, and a-IECs + a-IFs. Adult IFs derived from different donors are indicated with distinct numerical suffixes (e.g. a-IFs1 and a-IFs2).

First, we developed a co-culture model that contained human IECs and IFs of adult (a-IECs and a-IFs) and foetal (f-IECs and f-IFs) origin (Figure 2 and Supplementary Figure S1). Prior to co-culturing, IECs and IFs were cultured in different media formulations and characterised to confirm the retention of their specific phenotype under the new media conditions optimised for the model, referred to as intestinal mucosa medium (IMM). The characterisation of 3D organoid cultures and IEC monolayers has already been presented in our previous work (Roodsant et al., 2020; Stroulios et al., 2021). We performed a similar phenotypic characterisation of the IF cultures and confirmed that both a-IFs and f-IFs retain phenotype and ECM deposition capacity in IMM (Supplementary Figure S2). Once these qualitative controls were passed, IFs and IECs were sequentially seeded in direct contact on a standard cell culture insert and maintained in IMM (Figure 2A).

As IFs play a key role in ECM synthesis and organisation, we evaluated the effects of epithelial-mesenchymal crosstalk on the ECM microenvironment. The deposition of collagen type I and collagen type III, the predominant collagens in the intestinal lamina propria (Graham et al., 1988), was assessed by immunostaining (Figure 2B, Supplementary Figure S2D). Collagen types I and III were deposited in conditions including IFs, but not in IEC monocultures. In addition, only the co-cultures (a-IECs + a-IFs) formed a network of collagen bundles (Figure 2B). Gene expression analysis, performed on IEC and IF monocultures, corroborated the immunostaining data, revealing that both a-IFs and f-IFs contributed significantly (p < 0.001) to the ECM with higher levels of ECM-related genes (*COL1A1*, *COL3A1*, *COL4A1*, *FN1*, and *TNC*), compared to the respective IEC monocultures (Figure 2C, Supplementary Figure S1A). Interestingly, direct contact of IECs and IFs was necessary for the formation of the ECM network, as this was not formed when the IECs and IFs were cultured on opposite sides of the culture insert (Supplementary Figure S1B).

In addition to their role in ECM deposition, IFs are implicated in the formation of intestinal stem cell niches. IFs are known to secrete factors that promote epithelial proliferation and differentiation into various cell lineages (Brügger and Basler, 2023). Given that Paneth cells are an integral part of the stem cell niches and are essential for the stability and longevity of the intestinal mucosa (Mei et al., 2020), we quantified the number of Paneth cells in our IEC-IF co-culture model. On day 14, the number of Paneth cells was determined by counting Lysozyme (LYZ)- positive cells in IEC-IF co-cultures and IEC monocultures of adult origin (Figure 2D, Supplementary Figure S1F). Significantly (p < 0.001) higher numbers of Paneth cells were observed in the IEC-IF co-cultures (89.4 ± 19.1 and 51.1 ± 10.4 cells/mm^2^) compared to IEC monocultures (3.8 ± 3.3 and 2.1 ± 2.8 cells/mm^2^) (Figure 2D). Notably, while Paneth cells in IEC monolayers were difficult to detect due to their small size and low granularity, the LYZ+ granules of Paneth cells in IEC-IF co-cultures appeared larger and more numerous (Supplementary Figure S1F). RT-qPCR analysis of genes involved in Wnt, R-spondin, and BMP signalling, performed on monocultures, revealed that the increased presence of Paneth cells is likely supported by IFs expressing key markers of these signalling pathways. Significantly higher levels of *RSPO1*, *RSPO3*, *WNT5A*, *GREM1*, and *GREM2* were detected in both a-IFs and f-IFs, compared to a-IECs and f-IECs, respectively (Figure 2E, Supplementary Figure S1C). Consistent with the requirement for direct IEC-IF contact for ECM formation, the increase in Paneth cell count also correlated with direct IEC-IF co-culture. Although the administration of conditioned IMM from IF cultures (cond. IMM) to a-IEC monolayers resulted in a significant (p = 0.04) increase in the number of LYZ+ cells (16.5 ± 9.5 and 10.3 ± 2.8 cells/mm^2^) compared to a-IEC monolayers, this increase was still significantly (p < 0.0001) lower than that observed in direct IEC-IF co-cultures (Supplementary Figure S1D).

To determine whether the increased Paneth cell count enhanced the longevity of our cultures, we evaluated long-term cell survival by measuring trans-epithelial electrical resistance (TEER) every 2 days for 6 weeks (Figure 2F and Supplementary Figure S1E). At early time points (up to day 12), the presence of IFs in the co-culture did not significantly affect the barrier function of the intestinal mucosa model. However, in the long term, IFs appeared to enhance barrier stability. While the barrier integrity of IEC monolayers begins to deteriorate after day 30, IEC-IF co-cultures maintain TEER values above 200 Ω·cm^2^ for a longer period, up to day 44. Further, as Paneth cells are primarily found in the small intestine, but are rare in the colon and rectum (Yu et al., 2020), we investigated whether the ability of IFs to induce Paneth cells was restricted by the segment from which the IECs were isolated. To test this, we established colon-derived IEC-IF co-cultures (aC-IEFs + aC-IFs) and quantified the number of LYZ+ cells by immunostaining (Supplementary Figures S1G,H). In contrast to the increase of Paneth cell numbers in the small intestine-derived IEC-IF co-cultures, colon-derived IEC-IF co-cultures resulted in a small, but significant (p < 0.001), decrease in the number of Paneth cells (5.7 ± 2.6 and 8.0 ± 4.2 cells/mm^2^ in aC-IECs, 1.5 ± 1.3 and 1.3 ± 1.1 cells/mm^2^ in aC-IECs+aC-IFs). These findings indicate the recapitulation of segment-specific differences in the epithelial-mesenchymal interactions in our co-culture system.

To evaluate the regenerative capacity of our cultures, we performed wound-healing assays on 14-day IEC and IEC+IF monolayers (Supplementary Figure S3). Both systems exhibited progressive closure of the induced lesions within 72 h, as evidenced by imaging and recovery of transepithelial electrical resistance (TEER). Barrier function in both models returned to levels comparable to those of undamaged controls, confirming that IECs possess intrinsic regenerative potential after 14 days of culturing. Notably, however, the initial TEER drop was greater in the IEC+IF co-cultures, suggesting that the presence of fibroblasts promotes a more dynamic remodelling response to injury (Supplementary Figure S3B).

Collectively, these results highlight the critical roles of epithelial-mesenchymal crosstalk in orchestrating ECM synthesis and organisation, as well as in modulating intestinal epithelial barrier homeostasis.

### 2.2 Intestinal fibroblast–epithelial interactions regulate cytokine expression and modulate dendritic cell phenotype in an intestinal mucosa triple culture model

**FIGURE 3:**
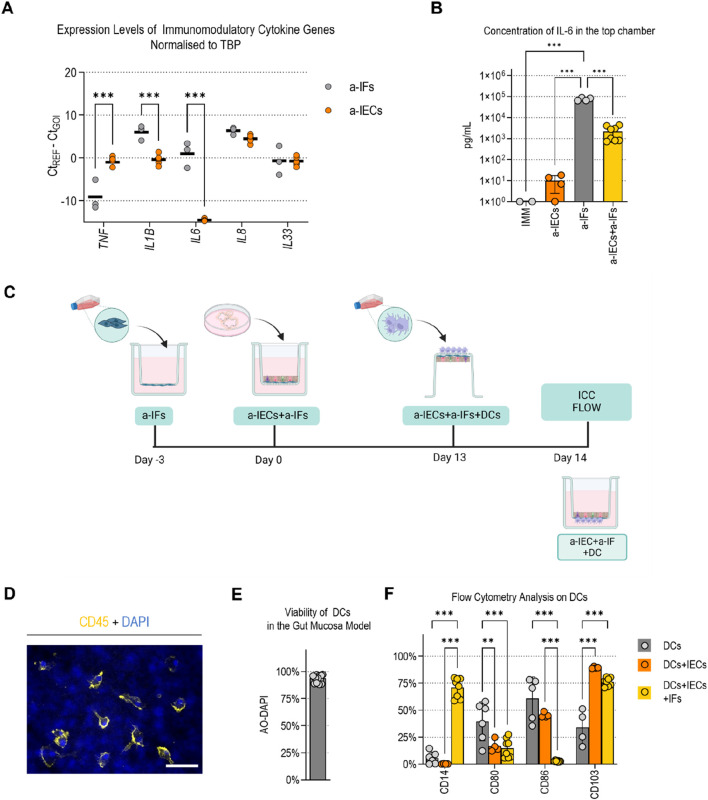
Epithelial-mesenchymal crosstalk and characterisation of DCs phenotype in intestinal mucosa triple-culture model. **(A)** RT-qPCR analysis of immunomodulatory cytokine genes performed on a-IECs and a-IFs cultures maintained for 14 days on cell culture inserts in IMM. Gene expression levels are plotted as -ΔCt values. Each point represents a biological replicate, orange and grey colours highlight data related to a-IFs (n = 3) and a-IECs (n = 5), respectively. Gene names are listed at the bottom of the plot. Statistical analysis employed a two-way ANOVA, along with Šídák’s multiple comparison test. **(B)** Bar graphs indicating the concentration of IL-6 secreted in the cell culture medium (IMM) by a-IF, a-IECs (n = 4), and a-IECs+a-IFs (n = 8) after 48 h. Each point indicates a biological replicate, and bars represent the mean and standard deviation. Statistical analysis employed a one-way ANOVA and Tukey’s multiple-comparison test. **(C)** Schematic representation of the workflow to establish a intestinal mucosa triple-culture model comprising a-IECs, a-IFs, and monocyte-derived DCs. **(D)** Representative immunofluorescence image at day 14 displaying CD45 expression in DCs attached to the basal side of the insert containing the IECs and IFs on the apical side (scale bar = 50 µm). **(E)** Cell viability assay based on acridin orange-DAPI stain performed at day 14 on DCs located in the basal chamber of the intestinal mucosa model maintained in IMM. Data points on the graph represent results from two independent experiments across eight biological replicates (n = 8), with bars representing the mean and standard deviation. **(F)** Flow cytometry analysis of CD14, CD80, and CD86 expression levels at day 14 in DCs cultured in the intestinal mucosa model in IMM. Each point represents a biological replicate (n = 6). The bars represent the mean with the standard deviation. Statistical analysis employed a two-way ANOVA and Tukey’s multiple-comparison test.

IFs perform several immunological functions under both homeostatic and inflammatory conditions (Thomson et al., 2020). Thus, to explore their immunomodulatory properties in our model, we conducted a gene expression analysis to assess the contribution of IFs and IECs to the transcription of some pro-inflammatory cytokine genes (Figure 3A). RT-qPCR analysis, performed on monolayers of IFs and IECs cultured in IMM, revealed that IECs expressed higher levels of *TNF* compared to IFs, which aligns with the function of TNFα in regulating epithelial cell turnover (Parker et al., 2019). In contrast, no differences were noted in the expression levels of *IL8* and *IL33*, while IFs expressed significantly (p < 0.001) higher levels of *IL1β* and *IL6*. To further investigate this at the protein level, we quantified secreted IL-6 in the supernatants of the different monocultures (Figure 3B). Consistent with the RT-qPCR data, high levels of IL-6 (75.2 ± 12.2 ng/ml) were measured in supernatants from a-IF cultures. Interestingly, the levels of secreted IL-6 in a-IECs+a-IFs (2.12 ± 1.5 ng/ml) were significantly lower (p < 0.001) compared to a-IF monocultures, suggesting that IEC-IF crosstalk may regulate IL-6 cytokine levels in the extracellular environment.

As IL-6 signalling was observed to be finely regulated in the IEC-IF co-cultures, and given the well-established role of IL-6 signalling in modulating intestinal DC function (Xu et al., 2022), we aimed to establish the first layers of the intestinal mucosa immune response by incorporating DCs into our IEC-IF *in vitro* model. The responsiveness of DCs to environmental cues, along with their antigen-presenting function, made them a desirable choice for a model incorporating early immune responses. Therefore, to further enhance phenotypical and functional relevance of our *in vitro* model, we co-cultured a-IECs and a-IFs with DCs for 48 h after IEC-IF co-culture establishment (day 14) (Figure 3C). The optimised media, IMM, used for IEC-IF co-culture was also verified to support DC phenotype and function prior to co-culture (Supplementary Figure S4).

DC phenotype, viability and functionality in the intestinal mucosa triple-culture model were thoroughly evaluated by immunostaining, flow cytometry, and ELISA (Figures 3D–F). Immunofluorescence microscopy verified the attachment of DCs to the basal side of the inserts with IECs and IFs on the apical side (Figure 3D). The presence of DCs on the basal side was evident by the detection of CD45, a pan-hematopoietic marker (Marone et al., 2023). Although the DCs were derived from a different donor than IECs and IFs, they were not activated, as demonstrated by a branched morphology and uniform dispersion. A cell viability assay, based on acridin orange (AO)-DAPI staining, performed on DCs harvested from the basal chamber of the intestinal mucosa model indicated that DCs remained highly viable (93.9% ± 3.2%) (Figure 3E).

To elucidate the immunomodulatory effects on the DCs in the co-cultures, the expression of CD14, CD80, CD86, and CD103 was evaluated by flow cytometry for different culture conditions [DC monoculture (DCs); IEC-DC co-culture (DCs+IECs); or IEC-IF-DC triple-culture (DCs+ECs+IFs)] (Figure 3F). These markers were chosen as IL-6 signalling has been previously associated with CD14 (Yao and Tseng, 2023) expression and modulation of CD80 and CD86 expression in intestinal DCs (Koorella et al., 2014), while CD103 is regarded as the primary marker expressed by resident intestinal DCs (Rua et al., 2011). The analysis revealed significant immunomodulatory effects on the DC phenotype in the triple-culture. Specifically, the triple culture (DCs+IECs+IFs) resulted in a significant (p < 0.001) increase in CD14 expression and a significant (p < 0.001) decrease in CD86 expression (p < 0.004) compared to the other conditions (DCs and DCs+IECs). Additionally, CD80 expression was significantly reduced (p < 0.001) when DCs were co-cultured (DCs+IECs and DCs+IECs+IFs), compared to DC monoculture. Moreover, the expression of the intestinal DC marker, CD103, was significantly increased (p < 0.001) in the co-cultures (DCs+IECs and DCs+IECs+IFs). These data suggest that co-culturing DCs with IECs and IFs leads to phenotypic modulation, promoting the acquisition of resident intestinal tolerogenic DC markers.

Overall, these results demonstrate the possibility of incorporating several layers of cellular complexity within the intestinal mucosa by co-culturing intestinal epithelial and mesenchymal cells with antigen-presenting dendritic cells. These data indicate that the IEC-IF co-culture model recapitulates key aspects of IF immunomodulation. Furthermore, our data suggest that the co-culture of peripheral blood-derived DCs within the IEC and IF microenvironment drives them towards an intestinal lineage, unlocking the potential of this *in vitro* model to study their cellular responses under different pathophysiological conditions.

### 2.3 Single-cell RNA sequencing identifies epithelial differentiation states, fibroblast subpopulations, and donor-specific differences in intestinal cultures

**FIGURE 4:**
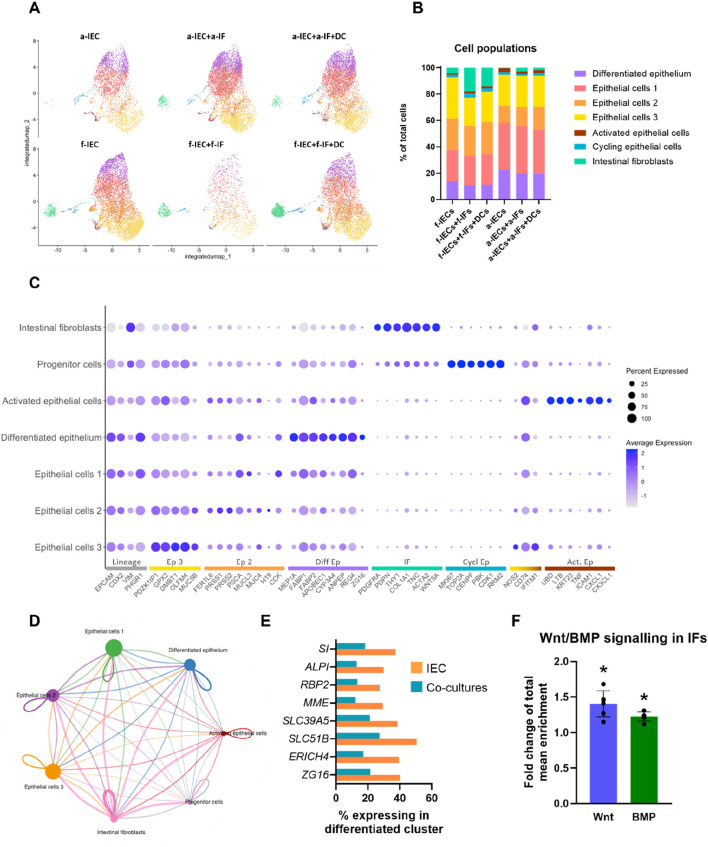
Single cell RNA sequencing analysis of foetal and adult IECs, IECs+IFs and IECs+IF+DCs cultures. **(A)** UMAP visualisations of the clusters, compiling differentiated epithelium, epithelial cells 1, epithelial cells 2, epithelial cells 3, activated epithelial cells, progenitor cells, and intestinal fibroblasts, in pooled foetal and adult IEC, IEC+IF and IEC+IF+DC samples compiling 28,528 total single cells and 37,493 genes, based on the expression of known marker genes after 15 days of culturing. **(B)** Relative proportions of cell clusters within foetal and adult IEC, IEC+IF and IEC+IF+DC cultures (biological n = 2, technical n = 6). **(C)** Dot plot of key markers for each cell cluster in the combined dataset (biological n = 6, technical n = 6). Values are scaled with 2 (blue) representing the highest, normalised expression of a given gene and 0 (white) representing the lowest normalised expression. Dot size indicates the percentage of total cells in a cluster that express the gene of interest. **(D)** Cell-cell communication network between cell types, with the width of the arrows representing the strength of the communication across all detected pathways. DE, differentiated epithelium; CE, cycling epithelial cells; AE, activated epithelial cells; IF, intestinal fibroblasts; E1, epithelial 1; E2, epithelial 2; E3, epithelial 3. **(E)** Expression of differentiation-associated epithelial markers between pooled IEC (biological n = 2, technical n = 6) and co-cultures (biological n = 4, technical n = 6) within the differentiated epithelium cluster. **(F)** Mean expression of Wnt and BMP signalling-associated genes in IFs, compiled and normalised to the mean enrichment of all analysed cells across all samples (biological n = 6, technical n = 6), with error bars representing SD. Statistical analysis was performed using a one-sample Wilcoxon test, and significance was accepted at p = 0.05.

To further investigate the cellular composition of our culture models and study the influence of co-cultures on the epithelial layer, we performed scRNA-seq on 28,528 cells and 37,493 genes across foetal and adult IEC, IEC-IF, and IEC-IF-DC culture conditions. We sampled the apical cell fraction of the intestinal cultures, excluding the basolaterally seeded DCs. Uniform manifold approximation and projection (UMAP) revealed seven major clusters representing intestinal fibroblasts, proliferative epithelial progenitors, immature epithelial populations, and differentiated epithelial subsets (Figures 4A–C). Cluster identities, viz., intestinal fibroblasts, differentiated epithelium, progenitor cells, activated epithelial cells, epithelial cells 1, epithelial cells 2, and epithelial cells 3, were assigned based on canonical markers (Supplementary Table S4).

As expected, intestinal fibroblasts ECM (e.g., *COL1A1*, *FN1*) and stromal genes (*THY1*, *VIM*), with subsets aligning to known fibroblast subtypes (*PDGFRA, ACTA2, FOXL1*). Cycling cells displayed high cell-cycle activity (*MKI67, TOP2A, RGMB, CDK1*), although we did not detect any expression of canonical stem cell markers *LGR5, ASCL2,* and *SMOC2*. Differentiated populations expressed both absorptive (*MEP1A, MTTP, APOBEC1, FAPB2)* and secretory markers (*REG4*, *ZG16*) (Figures 4A–C). Interestingly, foetal-derived cultures were enriched for goblet cell marker *ZG16*, while adult-derived cultures contained more *REG4* ^*+*^ secretory cells associated with deep crypt niches, which is a novel marker for enteroendocrine cells (EEC) (Supplementary Figures S5B,C) (Sasaki et al., 2016; Grun et al., 2015). *CCK* expression, which marks a specific EEC subset, was also elevated in adult samples across multiple clusters, indicating increased EECs presence in adult cultures (Supplementary Figures S5B,C). The existence of a transitional epithelial population (Epithelial cells 1) expressing differentiated markers to a lower extent suggests ongoing maturation (Figure 4C). The relative expression of *PHGR1* follows the predicted axis of differentiation in our samples, with the highest expression in differentiated epithelial cells and the lowest in progenitor cells (Figure 4C). Earlier research shows the association of increased *PHGR1* expression with epithelial differentiation, which aligns with our observations (Oltedal et al., 2018).

We also identified an epithelial cluster (Epithelial cells 3) (Figures 4B, C) with a transit-amplifying/stem cell gene signature (*OLMF4* ^*high*^) and expression of stress- and inflammation-associated transcripts (e.g., *UBD, IFITM1, NOS2)*. Along with the presence of absorptive differentiation markers (*PDZK1IP1, GPX2*) and secretory markers (*MUC5B, MUC4, DMBT1*), this cluster indicates a dynamic immature cell population that differentiates into both secretory and absorptive cells under oxidative stress or inflammatory stimuli (Figure 4C). Another population (Epithelial cells 2) (Figures 4A, B) expressed secretory markers (*FER1L6, PRSS1, MUC4 and MUC5B*) at higher levels, which suggests the presence of secretory epithelial cells (Figure 4C). Notably, classical markers of enteroendocrine, goblet, and Paneth cells were not robustly detected, suggesting either low abundance or technical under-representation.

Lastly, we distinguished a small cluster, comprising activated IECs engaged in inflammatory signalling, tissue remodelling, and repair processes (Figures 4A,B). These cells are marked by elevated expression of inflammatory genes (*LTB*, *TNF*, *UBD*) and chemoattractants (e.g., *CXCL1*, *CX3CL1*) (Figure 4C). This suggests the existence of a minor constitutive cellular stress or inflammatory cluster, irrespective of stimulation.

To validate our cluster annotations, we integrated our dataset with a published human intestinal scRNA-seq reference dataset (Fujii et al., 2018). Following integration, proliferative epithelial cells mapped predominantly to the TA1 and TA2 populations of the reference dataset, supporting their annotation as cycling epithelial cells. The activated epithelial cluster did not correspond to a canonical reference population and instead overlapped primarily with cells labelled as “Unknown”, suggesting that this cluster may represent a culture-specific activated epithelial state. The remaining epithelial clusters showed overlap with multiple differentiated epithelial populations, consistent with the existence of transitional differentiation states rather than discrete mature lineages. (Supplementary Figure S5A).

Besides cluster characterisation, extended observations can be appreciated after quantification of cluster composition, which revealed clear donor-dependent differences: adult cultures contained a higher fraction of differentiated/maturing epithelium (57.6% vs. 39.8% in foetal). In comparison, fibroblasts and secretory populations were proportionally enriched in foetal samples (27% vs. 15.2%) (Figure 4B).

To elucidate influence of additional cell types on epithelial differentiation, we assessed cell-cell communication using CellChat, where the intestinal fibroblast cluster displays the strongest outgoing communication signalling to other cell clusters (Figure 4D). This analysis depicts that ECM protein deposition is the predominant cell signalling from the intestinal fibroblasts (Supplementary Figures S5F,G). Furthermore, we analysed differences in expression of differentiated enterocyte markers (*SI, ALPI, RBP2, MME, SLC39A5, SLC51B, ERICH4*) and goblet cell marker *ZG16* of cells within the differentiated epithelium cluster between IEC monocultures and pooled co-cultures (Figure 4E). Across multiple markers, IEC samples show up to 2-fold more positive cells in the differentiated cell cluster than in the co-cultures. These data could point to increased terminal differentiation in IEC cultures compared to co-cultures, which could aid in explaining the previously described differences in culture longevity between culture conditions. Additionally, we investigated the influence of IFs on the Wnt and BMP signalling gradients in the intestine by quantifying Wnt- and BMP-associated gene expression (Figure 4F). The expression of these genes was compared between cells of pooled samples from the intestinal fibroblast cluster and the mean expression of all analysed cells, which resulted in a significantly higher enrichment of both Wnt and BMP pathways for IFs compared to other cells (p = 0.0312). These observations confirm the contribution of IFs to the Wnt/BMP differentiation gradient in our culture models, consistent with our previous data (Figures 2F,G).

Interestingly, cells in the foetal cycling cell cluster express mesenchymal/fibroblast-like markers in addition to cell-cycle genes, which are not detected in the adult samples (Supplementary Figures S5B,C). Surprisingly, the fibroblast-like cell signature was also detected in f-IEC cultures without exogenous IF addition. These data suggest the possibility of epithelial-mesenchymal transition, which can occur due to the plasticity of foetal stem cells, subsequently giving rise to fibroblasts in our culture conditions (Sprangers et al., 2021; Hahn et al., 2017) (Supplementary Figures S5D,E). Furthermore, the presence of this phenomenon could explain the enrichment of cells with a fibroblast signature in all three foetal samples, as compared to adult samples.

Together, these findings confirm that our system maintains the major cellular lineages of the human intestine, along with reveals age- and culture condition-dependent variation in epithelial maturation.

### 2.4 Dendritic cell and macrophage immune activation and phenotypic shift in response to pro-inflammatory stimuli in intestinal mucosa co-cultures

**FIGURE 5:**
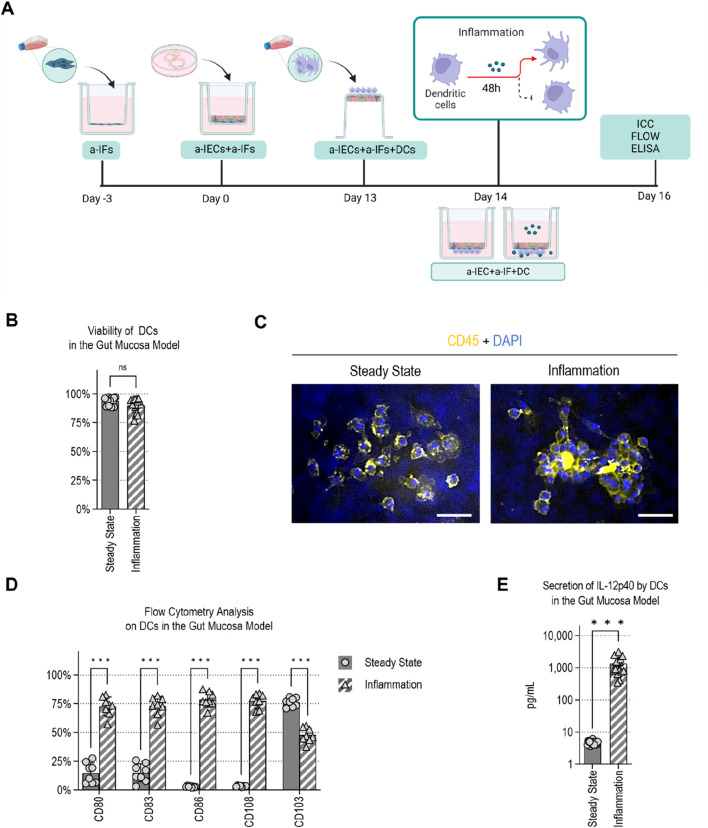
DCs remain functional in the intestinal mucosa triple-co-culture model. **(A)** Schematic representation of the workflow for modelling inflammation in the intestinal mucosa using the triple-culture model with DCs. **(B)** Cell viability assay based on acridin orange-DAPI stain performed at day 14 on DCs located in the basal chamber of the intestinal mucosa model maintained in steady state or inflammatory conditions. Data points on the graph represent results from two independent experiments across eight biological replicates (n = 8), with bars representing the mean and standard deviation. Statistical analysis employed a paired t-test. **(C)** Representative immunofluorescence images at day 14 displaying CD45 expression in DCs attached to the basal side of the insert, comparing steady state and inflammatory conditions (scale bars = 50 µm). **(D)** Flow cytometry analysis of CD80, CD83, CD86, CD108, and CD103 expression levels at day 14, comparing steady state and inflammatory conditions. Each point represents a biological replicate (n = 8). Statistical analysis employed a two-way ANOVA, along with Šídák’s multiple-comparison test. **(E)** Bar graphs indicating the concentration of IL-12p40 secreted in the cell culture medium by DCs in the intestinal mucosa model at day 14, comparing steady state and inflammatory conditions. Data points on the graph represent results from two independent experiments across 8 biological replicates (n = 8), with bars representing the mean and standard deviation. Statistical analysis employed a paired t-test.

As described in Figure 2, DCs were unaffected when cultured with allogenic IECs and IFs in steady state conditions. To verify that DCs could also maintain their function in the intestinal mucosa co-cultures, we induced an inflammatory condition for 48 h, using a commercially available cocktail of pro-inflammatory stimuli, and observed the response of the DCs (Figure 5A). Despite a slight, non-significant decrease in cell viability, DCs remained highly viable (89.9% ± 6.0%) even in the inflammatory cultures (Figure 5B). In contrast to the steady state condition, the inflammatory environment triggered the formation of tightly packed clusters of DCs at the basal side of the culture insert (Figure 5C). This change in cellular morphology and phenotype strongly suggested that DCs underwent immune activation. Flow cytometry analysis of surface marker expression and quantification of cytokine secretion, such as IL-12p40, serve as the primary readouts to assess DC activation (Bates et al., 2015). To correlate the observed morphological changes with DC activation, we assessed the expression of T-cell stimulatory receptors CD80 and CD86 (Lim et al., 2012), DC maturation markers CD83 and CD108 (Li et al., 2019; van Rijn et al., 2016), and CD103, a marker distinguishing tolerogenic intestinal DCs promoting homeostasis, from inflammatory DCs driving effector immune responses (Annacker et al., 2005). These markers were analysed by flow cytometry (Figure 5D) and complemented by quantifying IL-12p40 levels in the supernatant (Figure 5E). Compared to steady state cultures, DCs exhibited significantly (p < 0.0001) higher levels of CD80, CD83, CD86, and CD108 in response to pro-inflammatory stimuli. Concurrently, there was a significant (p < 0.0001) decrease in CD103 expression, indicating the acquisition of an activated intestinal DC phenotype capable of initiating an adaptive immune response. Lastly, levels of IL-12p40, detected in the supernatant *via* ELISA, were significantly (p value < 0.0001) higher in inflammation (1337.6 pg ± 797.5 pg) compared to steady state cultures (4.5 pg ± 0.5 pg).

In addition to DCs, we also optimised conditions to incorporate monocyte-derived MΦs into the intestinal mucosa model as a proof-of-concept, given their pivotal roles in intestinal homeostasis, inflammation, wound healing, and tumorigenesis (Lee et al., 1985). Distinct M1 and M2 macrophage subtypes (Strizova et al., 2023) were generated from non-activated MΦs (M0) *via* specific activation factors in triple-cultures comprising a-IECs, a-IFs, and MΦs under steady-state conditions for 48 h (Supplementary Figure S6A). Immunofluorescence confirmed MΦ attachment to the basal side of the culture insert (Supplementary Figure S6B), and AO-DAPI viability staining showed that all subtypes remained highly viable during co-culture (93.7% ± 4.0% M0; 95.4% ± 3.2% M1; 94.7% ± 4.4% M2) (Supplementary Figure S6C).

Polarisation into their respective activation states was confirmed with flow cytometry (Supplementary Figure S6D). While M0 MΦs showed low basal expression of M1 (CD80: 26.7% ± 10.6%; CD86: 33.8% ± 6.4%) and M2 (CD206: 20.27%; CD209: 35.91%) markers, M1 stimulation significantly upregulated CD80 (72.4% ± 14.6%) and CD86 (62.8% ± 17.0%), and M2 stimulation increased CD206 (76.4% ± 8.7%) and CD209 (88.2% ± 3.8%) expression.

These data indicate that our model also facilitates the incorporation of MΦs, effectively recapitulating MΦ-plasticity across steady-state, pro-inflammatory, and tissue-remodelling phenotypes.

Together, these results indicate that our complex intestinal mucosa model supports the culture of functional APCs and that retain the capacity to trigger an effective immune response when exposed to pro-inflammatory stimuli *in vitro*.

### 2.5 Stromal and immune cells contribute to viral infection dynamics in the intestinal mucosa culture model

Building upon this functional characterisation of DCs, we investigated how the inclusion of stromal and immune cells within the model influences viral infection dynamics. Mesenchymal cells like IFs are known to be targets for certain viruses, including cytomegalovirus (CMV), which exhibits broad cellular tropism (Sinzger et al., 1995). Given the gastrointestinal tract’s role as a major route of CMV infection (Yeh et al., 2024), we used our foetal-derived IEC–IF co-culture system to assess *in vitro* susceptibility to a clinical CMV isolate, CMV10 (Supplementary Figure S7A). Over 15 days, qPCR analysis revealed that viral replication occurred only in conditions containing f-IFs, not in f-IEC monocultures, suggesting that IFs are primary targets of CMV in the intestinal setting (Supplementary Figure S7A). CMV infection of f-IFs in the IEC-IF co-cultures was also confirmed by the co-localisation of immediate early antigen 1 (IE1) exclusively in EPCAM negative cells located beneath the epithelium (Supplementary Figure S8C). This infection pattern supports the idea that stromal cells can act as viral reservoirs or mediators of infection at the basal compartment of the intestinal epithelium.

Having established that both the stromal and immune compartments retain physiological functionality and susceptibility in our model, we examined their combined contribution to infection by a common enteric virus, Enterovirus A71 (EV-A71). Previously, we have shown that EV-A71 can infect IECs *in vitro*, with a higher infection efficiency when the virus was inoculated from the basolateral side of the culture insert compared to the apical side (Aknouch et al., 2023). In parallel, we have demonstrated that DCs are capable of transmitting EV-A71 to target cells, independent of viral replication (Helgers et al., 2022). Based on these previous findings, we hypothesised that EV-A71 infection of the intestinal mucosa can be better recapitulated *in vitro* using a more complex culture system containing APCs.

**FIGURE 6:**
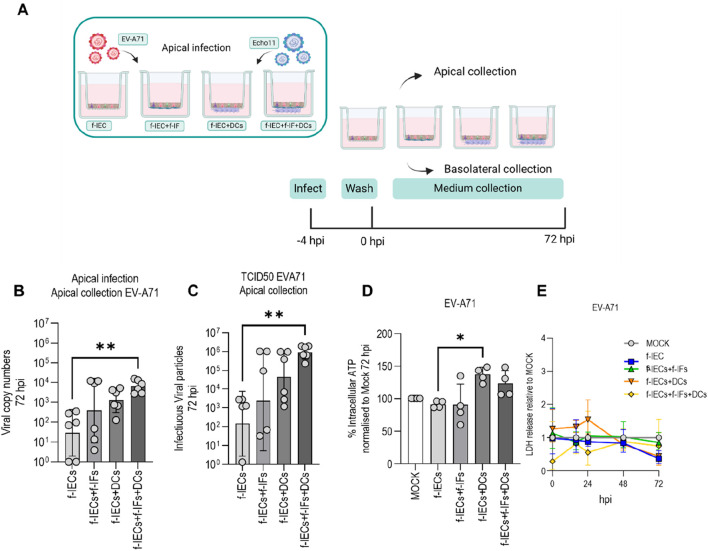
EV-A71 infection in foetal mono-cultures, co-cultures and intestinal mucosa model. **(A)** Schematic representation of viral inoculation and readout. **(B)** Average viral copy numbers and **(C)** infectious viral particles of EV-A71, 72 h post-infection (biological n = 2, technical n = 3, error bars = SD). Statistical analysis employed a Kruskal-Wallis test and Dunn’s multiple-comparison tests. **(D)** Average increase (%) of intracellular ATP indicative of metabolic activity (biological n = 2, technical n = 2, error bars = SD). Statistical analysis employed a Kruskal-Wallis test and Dunn’s multiple-comparison tests. **(E)** LDH release relative to the uninfected mock condition for all models over time (biological n = 2, technical n = 2, error bars = SD). Statistical analysis employed a Kruskal-Wallis test and Dunn’s multiple-comparison tests.

To test this hypothesis, we infected four culture types with varying levels of complexity (f-IECs, f-IECs+f-IFs, f-IECs+DCs, and f-IECs+f-IFs+DCs) with EV-A71 from the apical side (Figure 6A). We observed a significant increase in EV-A71 viral copies by RT-qPCR and a significant rise in infectious virus particles in f-IECs+f-IFs+DCs, compared to f-IECs (Figure 6B,C). Consistent with our previous reports, viral shedding was observed only on the apical side, with no viral RNA detected in the basal compartment. To rule out that the increase in viral copies was due to higher cell numbers, we also infected these cultures with Echovirus 11 (E11), which infects IECs *in vitro* upon apical infection. For E11, only a modest increase in copy numbers was detected in all the conditions, including DCs (f-IECs+DCs and f-IECs+f-IFs+DCs), compared to the conditions lacking them (f-IECs and f-IECs+f-IFs) (Supplementary Figure S8).

Finally, as an increase in metabolic activity has been associated with DC activation following pathogen recognition (Wang et al., 2022; Wu et al., 2023b), we measured the percentage of intracellular ATP in our cultures 72 h post infection. We observed a significant increase in metabolic activity following EV-A71 infection in all conditions that included DCs (f-IECs+DCs and f-IECs+f-IFs+DCs), compared with conditions that lacked them (f-IECs and f-IECs+f-IFs) (Figure 6D). This effect was replicated to a smaller extent within the E11 infected cultures, albeit not statistically significant (Supplementary Figure S8E). No significant (p > 0.05) decrease in cell viability following EV-A71 or E11 infection was observed in any condition by LDH assay (Figure 6E, Supplementary Figure S8F).

In summary, these results demonstrate that mesenchymal cells and DCs play distinct yet complementary roles in mediating viral infection in our intestinal *in vitro* model. The infection of IFs by CMV underscores the susceptibility of the stromal compartment, while the inclusion of DCs effectively mimics the dynamic interplay between DCs and IECs during enterovirus infection. Together, these findings highlight how increasing the cellular complexity of the intestinal model enhances its capacity to replicate human viral infection dynamics and immune responses *in vitro*.

## 3 Discussion

Recently, OoC systems have been developed that elegantly recapitulate important features of the intestinal mucosa, but barriers such as high costs, lack of standardisation, the need for special equipment, and a steep learning curve limit their widespread use. To overcome this, while balancing the simplicity of organoids and the complexity of OoC systems, we undertook a knowledge-guided and easily reproducible assembly of a complex intestinal mucosa model on widely used cell culture inserts. This resulted in the establishment of a modular *in vitro* co-culture model with regenerative capabilities that recapitulates the complexity of the intestinal mucosa, including epithelial, mesenchymal, and immune cells. We optimised culture conditions and protocols to make the model robust, reproducible, and easy to use. We showed that it can be easily adjusted to comprise a variety of cell types in different combinations and adapted to specific scientific questions. Additionally, we provided evidence of the value of a primary human intestinal mucosa model for studying the multicellular crosstalk necessary for maintaining homeostasis of the intestinal barrier, and its role in disease, inflammation and viral infections.

In the intestine, IFs are responsible for ECM deposition to support the pericellular matrix and basal membrane of epithelial cells (Vilardi et al., 2024). In this study, we describe the importance of direct contact between IFs and IECs to achieve robust ECM deposition and organisation (Thibeaux et al., 2012; Maimets et al., 2022)*.* Additionally, this close crosstalk promotes the differentiation of Paneth cells, which are essential cellular components of the stem cell niche and contribute towards tissue regeneration (Ramadan et al., 2022; Duckworth, 2021; Farin et al., 2012). Interestingly, in our model, Paneth cell differentiation was promoted only in the context of the small intestine, with no similar effect observed when modelling the colonic epithelium. These *in vitro* findings align with the study by Hickey and colleagues (Hickey et al., 2023) who, using single-cell RNA sequencing of human tissue, showed that Paneth cells specifically populate the small intestine and organise into specific “neighbourhoods”. They postulated that such functional neighbourhoods across the intestinal tissue define the cellular composition and function of the human intestine (Hickey et al., 2023). Similarly, in an *in vivo* study performed using murine models, Maimets and colleagues (Maimets et al., 2022) found that the mesenchyme plays a critical role in defining the regional identity of the intestinal epithelium by modulating the Wnt signalling. The fact that such segment-specific differences can now be recapitulated in a cell culture system such as the one described here, represents a valuable contribution to increase further the predictive value of results obtained using *in vitro* models.

Beyond structural organisation, the interplay between epithelial and fibroblast compartments also regulates immunological signalling within the intestinal niche. *In vivo,* IFs interact with resident immune cells through the secretion of pro-inflammatory cytokines such as TNFα, IL-1β, IL-6, IL-8, and IL-33 (Cavagnero and Gallo, 2022; Pasztoi and Ohnmacht, 2022). Through the tight regulation of the levels of secretion of these cytokines, the IFs immune modulate APC functions under both homeostatic and inflammatory conditions (Thomson et al., 2020). In our co-culture system, we observed that the interaction between IFs and IECs influences a number of these cytokines, including IL-6. This cytokine is of particular interest as it regulates epithelial proliferation, ECM remodelling, and most prominently, immune cell activation (Jeffery et al., 2017; West, 2019; Barnes et al., 2011). Interestingly, IL-6 dysregulation has been linked to inflammatory bowel diseases (IBD), which is marked by chronic inflammation that fuels the development of intestinal fibrosis (Welz and Aden, 2023). For this reason, IL-6 has recently become a therapeutic target for intestinal pathologies (Shahini and Shahini, 2023; Schreiber et al., 2021; Ito et al., 2004; Danese et al., 2019). Importantly, clinical and histological evidence indicates that IBD flares tend to recur in the same anatomical regions of the intestine, such as identical ileal strictures or rectosigmoid segments, suggesting a form of site-specific inflammatory memory. In this context, residual intestinal fibroblasts, generally regarded as long-lived connective tissue cells, are thought to play a critical role, as fibroblast activation is repeatedly observed in these recurrently inflamed regions (Li et al., 2025). Unlike current *in vitro* models that often neglect fibrosis by focusing solely on epithelial barrier disruption induced by biochemical pro-inflammatory stimuli (Jung and Kim, 2022), our triple-culture model incorporates epithelial, mesenchymal, and immune cells. This allows for ECM synthesis and the potential to induce fibrosis *in vitro* via co-culture with M1 macrophages. In this context, we believe that our complex intestinal mucosa model could serve as a new screening platform where levels of IL-6 can be easily measured and targeted by compounds, and the effects of this cytokine can be readily analysed. We believe this feature makes it an ideal platform for comprehensively investigating IBD mechanisms and evaluating potential therapeutic interventions.

While fibroblasts serve as key mediators of epithelial maintenance and inflammatory balance, the addition of immune cells to the system revealed further layers of complexity in cellular communication. Moreover, in establishing this *in vitro* model, we also observed that the cross-talk between different cell types and the environment influences cell identity. In particular, dendritic cells demonstrated high sensitivity to co-culture environments and exerted significant effects on cytokine secretion. In addition, DCs acquired a phenotype that closely resembles resident intestinal tolerogenic DCs, both in homeostatic (Beitnes et al., 2011; Rescigno and Di Sabatino, 2009) and inflammatory conditions (Dillon et al., 2010; Matsuno et al., 2017) as described *in vivo*.

To gain deeper insights into the cellular diversity and specialisation within these co-cultures, we complemented functional observations with transcriptomic profiling using single-cell RNA sequencing. Through analysis of the different culture types (IECs, IECs + IFs, IECs + IFs + DCs), we confirmed the presence of the most common cell types in the human intestine, such as cycling epithelial cells, absorptive enterocytes, and secretory epithelial cells. Although we found markers indicating the presence of goblet cells (*ZG16*), enteroendocrine cells (*REG4, CCK*), and Paneth cells (PRSS2), specific canonical lineage markers were undetected in our scRNA-seq dataset, hindering a particular clustering of specialised epithelial cell types. Although data in this study, together with earlier published qPCR and immunostaining show LGR5, LYZ, CHGA and MUC2 expression in our enteroid-derived model (Roodsant et al., 2020; García-Rodríguez et al., 2021), technical aspects of sample preparation and the inherently low or variable expression levels of these transcripts in single cells could explain the observed results (Holloway et al., 2021; Moerkens et al., 2024; Yan et al., 2022; Ivich et al., 2025). *LGR5* transcripts are known to have low abundance and are dynamically expressed (Moerkens et al., 2024; Jadhav et al., 2017), whereas *LYZ* and *MUC2* transcripts are often lost during dissociation due to the fragility and plasticity within these rare secretory cell types (Haber et al., 2017; Beumer and Clevers, 2021). Together, these factors can explain the limited transcript detection of some canonical lineage markers in our scRNA-seq analysis, compared to those performed using qPCR or protein-based assays. Along with the general characterisation, we found indications of increased epithelial differentiation in IEC monocultures compared with IEC-IF(-DC) co-cultures and demonstrated the contribution of IFs to ECM and Wnt-BMP signalling pathways. In conjunction, these data indicate the potential importance of IFs in cell signalling and differentiation in our culture system, aligning with other data in our study and previously published work (Maimets et al., 2022). Additionally, the apparent presence of mesenchymal progenitor cells in foetal cultures, regardless of exogenous addition, may reflect the plasticity of foetal stem cells. Epithelial-mesenchymal transition could occur under specific culture conditions, subsequently giving rise to fibroblast-like cells after differentiation on culture inserts (Sprangers et al., 2021; Hahn et al., 2017).

After establishing the cellular composition and intrinsic heterogeneity of the co-culture, we evaluated the functional responsiveness of the model to viral infections. Other studies employing unpolarised *in vitro* intestinal models with limited success in supporting effective viral replication (Maidji et al., 2017), our highly polarised IF-IEC co-culture system demonstrated susceptibility to CMV infection. CMV targeted the IFs, similar to *in vivo* infections (Sinzger et al., 1995), enabling efficient *in vitro* replication of a clinical CMV strain, and indicating the potential of this co-culture system for virus pathogenesis studies.

It is well established that DCs play an essential role in triggering the initial innate immune response to viral infections in the intestine (Marongiu et al., 2021; Luis Muñoz-Carrillo et al., 2021). Previously, we have shown the susceptibility of *in vitro* polarised intestinal monolayers to EV-A71 infection, in which viral replication was primarily detected upon inoculation of the virus from the basal side of the epithelial cells (Aknouch et al., 2023). However, EV-A71, similar to other picornaviruses, is transmitted through the oral-faecal route (Huang et al., 2020) and therefore, is likely to interact with the gastrointestinal epithelium from the apical side. Our previous results, alongside the known ability of DCs to transmit the virus to cells downstream (Helgers et al., 2022), led us to hypothesise that EV-A71 infection could occur *via* a “Trojan horse” mechanism, involving the subversion of DC APC function. However, further mechanistic studies should be performed to fully determine the role of DCs in virus transfer within the complex intestinal setting. Infection of our intestinal mucosa model with EV-A71 allowed us to better mimic viral replication in the presence of DCs, with the virus inoculated from the apical side and viral replication detected at levels previously reported (Aknouch et al., 2023) only basolaterally. Interestingly, several other viruses, including poliovirus and norovirus, similarly bypass the intestinal barrier and initiate infection basolaterally (Siciński et al., 1990; Ouzilou et al., 2002; Hassan and Baldridge, 2019). In light of this, we believe our model has significant potential to recapitulate the molecular mechanisms of various viruses, many of which remain poorly understood.

Building on this capacity to capture immune-epithelial interactions, we expanded the model to include MΦs, which effectively replicated steady-state, pro-inflammatory, and tissue remodelling characteristics (Jiang et al., 2023). This optimised model more closely approximates key aspects of the intestinal microenvironment *in vivo* and provides a modular platform for investigating macrophage behaviour and interactions with multiple cell types.

Overall, with our co-culture model, we have provided an advancement in cellular complexity over the current *in vitro* intestinal models while ensuring their accessibility and enhanced physiological relevance in a widely used culture platform, such as the cell culture inserts. Using this approach, we recapitulated the crosstalk between multiple cell types and the first key elements of the intestinal mucosa, including intrinsic and innate immunity. We believe that our advanced intestinal mucosa model can already enhance the physiological relevance of current culture models. Furthermore, with the possible future implementation of microfluidics systems, this model has the potential to advance the current state of the art in studying human intestinal health and disease.

## 4 Study limitations

In this study, our focus was on introducing cellular diversity and specialisation by incorporating the first layer of the human intestinal mucosa. However, our model does not capture the full complexity of the mucosa and lacks other key cell populations. These include, but are not limited to, M cells, tuft cells, B-cells, and T-cells. Incorporating these cells will require even more refined culturing conditions and a completely isogenic system. Another key component of the intestinal mucosa currently missing from our model is the microbiome. However, we and others have demonstrated the feasibility of incorporating commensals into organotypic models, and similar approaches will further expand the physiological relevance of intestinal mucosa models (Nanlohy et al., 2024; Shah et al., 2016; Jalili-Firoozinezhad et al., 2019). Additionally, the described models in this study are not isogenic, which could have an influence on the interactions between the cell types and subsequent functional readouts. Future models should incorporate induced pluripotent stem cells (iPSCs) as sources for the different cell populations, which would result in a fully isogenic culture setting. Finally, not all experiments are conducted utilising the same donor configuration. Although we show broad applicability with both foetal and adult donors, the heterogeneity between donor ages can influence the results of infection modelling, as infant intestinal epithelium has a lower baseline and blunted inducible type-I/III interferon response, and immature efflux-transporter expression, compared with adult enteroids. These differences can alter IFN responses and influence viral dynamics in the mucosa model of adult and foetal donors (Adeniyi-Ipadeola et al., 2024).

## 5 Materials and methods

### 5.1 Cells and equipment

Unless otherwise stated, all cells, media, media supplements and reagents were provided by STEMCELL Technologies (Canada); plasticware was purchased from Starlab (United Kingdom); 6.5 mm cell culture inserts made of polyethylene terephthalate (PET), with a pore size of either 0.4 µm or 3 µm, and purchased from CellQART (Germany) or Greiner Bio-One (United Kingdom), and qPCR primers were purchased from Integrated DNA Technologies (IDT, Belgium). Colon fibroblasts (ATCC, cat# CRL-1459) and small intestinal fibroblasts (Innoprot, cat# P10760) were purchased from ATCC (United States) and 2B Scientific (United Kingdom) respectively.

### 5.2 Cell isolation

#### 5.2.1 Ethics statement

Human foetal intestinal tissue, gestational age 16–18United Kingdomweeks, was obtained from a private clinic by the HIS Mouse Facility of the Amsterdam University Medical Center. All donors supplied written informed consents for the use of foetal material for research purposes. These consent forms are kept at the clinic and the information available to the Amsterdam UMC does not allow identification of the donor without disproportionate efforts. The use of the anonymised material for medical research purposes is covered by Dutch law (Wet foetaal weefsel and Article 467 of Behandelingsovereenkomst).

#### 5.2.2 Generation of human foetal intestinal organoid cultures

For the generation of foetal small intestinal organoids, crypts were isolated from foetal intestinal tissue as described previously (Roodsant et al., 2020). Isolated crypts were suspended in Matrigel, dispensed in three 10 μl droplets per well in a 24-well tissue culture plate and covered with 500 μl medium. Enteroid cultures were routinely maintained at 37 °C, 5% CO_2_, and 95% humidity in Human IntestiCult™ Organoid Growth Medium Human (IntestiCult OGMh) supplemented with 100 U/mL penicillin/streptomycin (Pen-Strep) (Lonza). Medium was replenished every second day, and organoids passaged every 3-5 days.

#### 5.2.3 Generation and maintenance of human foetal intestinal fibroblast cultures

Following crypt isolation, tissue pieces were further digested in 1mg/mL Collagenase I for 45 min at 37 °C. Collagenase activity was inhibited by 0.05 mM EDTA, and the resulting cell suspension was passed through a 70 µm cell strainer. The collected cell suspension was centrifuged at 300 xg for 5 min, the supernatant discarded, and the cells resuspended in advanced DMEM/F12 (AdvDMEM/F12) (Gibco) supplemented with 100 U/mL (v/v) Pen-Strep, 1% (v/v) Glutamax and 2% (v/v) heat-inactivated foetal bovine serum (FBS, Sigma-Aldrich) (2% AdvDMEM/F12). The Primary human intestinal fibroblasts were allowed to adhere to the culture flask overnight and the medium was replenished thereafter. Intestinal fibroblasts were then routinely cultured at 37 °C, 5% CO_2_ and 95% humidity in 2% AdvDMEM/F12. The culture medium was replenished every second day and cells were subcultured by trypsinization upon reaching 70% confluence.

### 5.3 *In vitro* cultures

#### 5.3.1 Maintenance of adult human intestinal fibroblasts

Intestinal fibroblasts (IFs) derived from small intestine were purchased from 2B Scientific (cat #P10760). Colon fibroblasts were purchased from ATCC (cat #CRL-1459). IFs were expanded using the MesenCult™ Proliferation Kit on standard cell culture-treated flasks, coated with Animal Component-Free Cell Attachment Substrate (ACF-CAS). Cryopreserved IFs were plated at a density of 1 x 10^3 cells/cm^2^ in complete MesenCult™ Medium and incubated at 37 °C until 90% confluent. The culture medium was replenished every second day and cells were subcultured by trypsinization upon reaching 70% confluence.

#### 5.3.2 Maintenance of adult and foetal human intestinal organoids

Intestinal organoid cultures were maintained and expanded using IntestiCult OGMh in 10 µL Matrigel® as previously described (Aknouch et al., 2023), and according to the manufacturing protocol. The medium was changed every other day and the organoids were passaged every 7 days.

#### 5.3.3 Differentiation of monocytes into dendritic cells

Human Peripheral Blood Monocytes, either cryopreserved (cat #70034) or freshly isolated Human Peripheral Blood Leukopak (#70500), were differentiated into dendritic cells (DCs) using ImmunoCult™ Dendritic Cell Culture Kit (ImmunoCult DC), following the manufacturer’s instructions.

#### 5.3.4 Differentiation of monocytes into macrophages

Human monocytes, either cryopreserved or freshly isolated from whole blood, were differentiated into macrophages using ImmunoCult™ SF Macrophage Differentiation Medium (ImmunoCult MΦ), following the manufacturer’s instructions.

#### 5.3.5 Establishment of the advanced intestinal mucosa model comprising intestinal epithelial cells, intestinal fibroblasts, and antigen-presenting cells

The intestinal mucosa model, which includes IECs, IFs, and APCs, is established using a modular workflow, where each cell type is sequentially added to standard cell culture inserts. Alternatively, intermediate models comprising only two cell types (IEC-IF or IEC-APC) were also developed. All models were established using a single co-culture medium, IntestiCult™ Organoid Differentiation Medium Human (IntestiCult ODMh), referred to as IMM, from the moment the cells are seeded on the inserts. Briefly, on day −3, cell culture inserts were functionalized with 100 µL ACF-CAS for 1-h, followed by seeding either adult or foetal IFs at a density of 1 x 10^5^/cm^2^ in 300 µL IntestiCult ODMh in the apical chamber of the cell culture inserts. To the basal chamber 700 µL IntestiCult ODMh and IFs incubated at 37 °C until confluent. On day 0, adult or foetal intestinal organoids were mechanically and enzymatically fragmented into a single-cell suspension. The resulting IECs were then plated at a density of 7.5 x 10^5^/cm^2^ onto the confluent adult or foetal IFs in the apical chamber of cell culture inserts. IEC-IF co-cultures were maintained in IntestiCult ODMh. Medium was changed daily in the apical chamber, and every other day in the basal chamber. On day 14, APCs were harvested, inserts containing IEC-IF co-cultures were prepared by removing the medium and inverting them on an extra-depth plate. Subsequently, APCs were seeded onto the basal side of these inserts at a density of 1 x 10^5^/cm^2^ and allowed to attach at 37 °C for at least 3 h. Inserts were then returned to the upright position in tissue culture-treated plates, with 300 µL and 700 µL of IntestiCult ODMh in the apical and basal chambers, respectively. To ensure the IEC-IF co-cultures were ready for downstream applications, the integrity of the intestinal barrier was verified by (i) TEER measurements, (ii) paracellular permeability assay, and (iii) visualisation of a uniform network of tight junctions by ZO-1 immunostaining.

### 5.4 Single cell RNA sequencing

#### 5.4.1 Culturing and sample preparation

For this analysis, we cultured 2.5*10^6^ IECs together with 3.3*10^4^ IFs in the corresponding culture conditions of both foetal and adult donors for 14 days at 37 °C, 5% CO_2_ and 95% humidity in IntestiCult ODMh, after which the 10^5^ DCs were seeded on the basal face of the designated cultures and incubated for another 24 h. Subsequently, cells of the apical side of each culture were manually dissociated from culture inserts with 0.5 M EDTA (Invitrogen) and Accutase (Stemcell Technologies). Subsequently, samples were further dissociated into individual single-cell suspensions using TrypLE Express (Gibco), DNase I (Stemcell Technologies) and RNAsin (Promega). Cells were counted and viability was determined with BioRad automatic cell counter. Highly viable single cell suspensions were labelled using the CLindex® Sample Multiplexing Kit 16-Plex S (Singleron Biotechnologies, cat. no. 1050064) according to the manufacturer’s instructions. Single-cell suspensions were prepared and aliquoted (1–2 × 10^6^ cells per reaction) and individually labelled with distinct CLindex sample indices (Tags 1–6). Labelling reactions were performed at room temperature for 15 min in a thermomixer at 1000 rpm using the provided Labelling Solution and corresponding CLindex Sample Index reagents. Reactions were quenched for 5 min using the supplied Quencher Mix 1 and 2, followed by two washes in 1× PBS. Labelled cells were resuspended in 1× PBS, counted, and pooled in equal proportions before loading on the SCOPE-chip HD. Three CLindex tags were combined per chip (foetal cultures and adult cultures respectively), targeting approximately 30 000 cells captured (≈ 10 000 cells per tag).

#### 5.4.2 Single-cell capture and cDNA generation

Labelled cell suspensions (≈ 30 000 cells total per chip) were loaded onto GEXSCOPE® Single Cell RNA Library Kit HD V2 microfluidic chips (Singleron Biotechnologies, cat. no. 4180031). Individual cells were partitioned into microwells containing paramagnetic barcoded beads carrying oligo-dT primers, UMIs, and cell barcodes. After cell lysis, released mRNA molecules hybridized to bead-bound oligos. Beads were retrieved, washed, and subjected to reverse transcription at 42 °C for 90 min to generate barcoded cDNA, followed by PCR amplification. Amplified cDNA was purified using AMPure XP beads and quantified with a Qubit 4 fluorometer. Fragment size distribution was verified on an Agilent Fragment Analyzer 5200, where the main cDNA peak ranged between 200 and 5 000 bp (expected > 1 200 bp). Purified cDNA was shipped to Singleron Biotechnologies for library preparation and sequencing.

#### 5.4.3 Library preparation and sequencing

Transcriptome libraries were generated from 50 ng of amplified cDNA using the GEXSCOPE® Single Cell RNA Library Kit HD V2 workflow. cDNA was enzymatically fragmented, end-repaired, A-tailed, and ligated to truncated Illumina adapters. Adapter-ligated fragments were PCR-indexed with unique dual indices (UDI Set A) and purified by dual size selection (AMPure XP beads). Library QC confirmed an average insert size of 400–700 bp, with fragments < 300 bp representing < 5% of total molecules. In parallel, CLindex tag libraries were prepared from the amplified CLindex products obtained during cDNA amplification using the CLindex Primer 1 and CLindex Enrichment Mixes 1–4 according to the kit instructions. After enrichment PCR and purification, the CLindex libraries displayed a major fragment peak around ∼270 bp. All libraries were quantified with a Qubit 4 Fluorometer (Thermo Fisher Scientific) and validated by fragment analysis on an Agilent Fragment Analyzer 5200 system (Agilent Technologies) before sequencing. Equimolar transcriptome and CLindex libraries were pooled and sequenced on an Illumina NovaSeq X platform using paired-end 2 × 150 bp reads. Transcriptome libraries were sequenced to ≈ 400 Gb (total ≈ 45 000 read pairs per cell), and CLindex tag libraries to ≈ 50 Gb (≈ 5 000 read pairs per cell). Demultiplexing was performed using Illumina BaseSpace/BaseCloud software.

#### 5.4.4 Data processing and analysis

Raw sequencing reads were processed into gene-cell count matrices using CeleScope v 2.4.0 (Singleron Biotechnologies; www.github.com/singleron-RD/CeleScope). Barcode recognition, correction, and chemistry identification were executed automatically. Reads were aligned with STAR solo v 2.7.11a against the Ensembl 99 human genome (GRCh38). For multiplexing analysis, single-cell raw data were processed in CeleScope v 2.4.0 using the sequences of the corresponding CLindex tags and the associated single-cell transcriptome matrix. Cell calling and downstream QC were performed in R (v. 4.0.5) using Seurat (v. 4.0.5).

Each Adult and Foetal count tables were loaded individually into R version 4.2.2. and processed by Seurat v5.2.1. Cells with >10% mitochondrial gene expression or fewer than 2,000 detected genes were excluded. To remove potential doublets and low-quality cells, cells with >10,000 detected genes were also excluded. The filtered data were then normalised and scaled independently for each sample. Genes expressed in fewer than three cells across the dataset were removed. The resulting count matrix comprised 18,772 genes and 27,697 cells derived from six samples representing adult and foetal IEC, IEC+IF and IEC+IF+DC culture conditions.

To reduce donor-specific technical variation, each sample was first SCTransformed independently and subsequently integrated using the Reciprocal PCA (RPCA) workflow implemented in Seurat v5. RPCA integration identifies shared cellular states across samples by detecting mutual nearest-neighbour anchors and aligns cells with similar transcriptional profiles while preserving biological differences. Following integration, dimensionality reduction was recomputed using PCA and UMAP. Visual inspection of the integrated embedding demonstrated extensive mixing of cells from different donors and culture conditions, indicating effective mitigation of donor-driven batch effects while retaining biologically meaningful variation (Supplementary Figure S9). All downstream clustering and annotation analyses were performed on this integrated dataset.

#### 5.4.5 Cluster determination

We divided the combined dataset into 7 clusters using the Leiden algorithm implemented in Seurat’s FindCluster function. The clustering resolution was systematically varied until the desired number of clusters was achieved. The number of clusters was determined by examination of UMAP structure, silhouette score and graph modularity. The mean silhouette score quantifies transcriptional separation between clusters, and the graph modularity score measures community structure within the shared nearest-neighbour graph. Both metrics indicated that a solution with six clusters provided the optimal balance between separation and cohesion (Supplementary Figure S10).

However, visual inspection and biological interpretability suggested that one of the major clusters contained two transcriptionally distinct subpopulations. To better capture this structure, we selected a resolution that yielded seven clusters, effectively subdividing this large population while preserving the overall stability of the clustering solution.

#### 5.4.6 Cell type annotation

Cells were clustered using the Leiden community detection algorithm implemented in Seurat’s FindClusters function. Clustering resolution was systematically adjusted and evaluated to identify a biologically meaningful partitioning of the dataset. Differential gene expression analysis was performed using FindAllMarkers, and the top marker genes for each cluster were examined. Cell-type annotation was guided by canonical intestinal cell-type markers retrieved from previously published single-cell RNA-seq studies of the human intestine, including markers for major epithelial lineages as well as stem/progenitor and non-epithelial compartments. Cell-type labels were assigned based on concordance between cluster-enriched markers and published intestinal cell-type signatures (Supplementary Table S4).

#### 5.4.7 Differential gene expression

We performed differential gene expression using the Wilcoxon rank-sum AUC statistic implemented in the presto package (function *wilcoxauc*). For each cluster, genes were ranked according to a composite specificity score that combined (i) the area under the ROC curve (AUC), reflecting how well a gene distinguishes the target cluster from all other cells, and (ii) the difference in detection rate between the cluster and all remaining cells. The final score was defined as:

$$\text{score}=0.7\times \text{AUC}+0.3\times \left(\Delta \text{pct}\right),$$

where $\Delta \text{pct}={\text{pct}}_{\text{cluster}}-{\text{pct}}_{\text{others}}$ .

For each cluster, the top 10 genes by this composite score were retained as the most informative marker genes.

#### 5.4.8 Cell-cell communication analysis

Cell-cell communication between foetal and adult intestinal cell types was inferred using CellChat (v. 2.2.0) package in R. The sc-RNA seq data was log-normalized and compared to the CellChatDB.human database to detect potential significant signalling pathways and sender/reciever cell types. The inference of intercellular interaction networks was performed using default parameters. More information can be found on the official CellChat v2 Github https://github.com/sqjin/CellChat (Jin et al., 2025).

### 5.5 Viruses

#### 5.5.1 Propagation of enterovirus A-71 and echovirus 11

Human rhabdomyosarcoma (RD) cells (CCL-136™ ATCC) and African green monkey kidney (Vero) cells (CCL-81™ ATCC), were cultured in Eagle’s minimum essential medium (EMEM) (Lonza) supplemented with 8% FBS, 100 U/mL Pen-Strep, 0.1% (v/v) L-glutamine (Lonza), and 1% (v/v) non-essential amino acids (100x) (ScienceCell Research Laboratories). Cell lines were routinely cultured at 37 °C with 5% CO_2_, 95% humidity and passaged every 7 days using 0.05% (v/v) Trypsin/EDTA (Gibco). EV-A71 C1-91-480 obtained from the RIVM (GenBank: AB552982.1) and E11 was propagated in RD and Vero cells respectively.

#### 5.5.2 Propagation of cytomegalovirus

Human embryonic lung fibroblast (HEL) cells (isolated at Amsterdam UMC) were cultured in EMEM supplemented with 8% FBS, 100 U/mL pen-step, 0.1% (v/v) L-glutamine and 1% (v/v) non-essential amino acids at 37 °C with 5% CO2. A clinical isolate of CMV was derived from patient urine material (isolated at Amsterdam UMC) and propagated in HEL cells and retinal pigment epithelial cells (ARPE-19).

### 5.6 *In vitro* modelling of viral infections

#### 5.6.1 Viral infection with EV-A71 and E11

Viral infections on foetal HIE, HIE+IFs, HIE+DCs and HIE+IFs+DCs cultures were performed as described previously (García-Rodríguez et al., 2024; Aknouch et al., 2022). Briefly, viral stocks (EV-A71 and E11) were diluted to 10^5^ TCID50/50 μL in IntestiCult ODMh. Cultures were apically inoculated with virus, by removing 50 μL of medium and adding 50 μL of viral inoculum. The cultures with virus were incubated for 4 h at 37 °C with 5% CO_2_. Following incubation, unbound virus was washed away from the apical compartment with advDMEM/F12 three times. Inserts were incubated for 10 min with IntestiCult ODMh after which the 0 h post infection (hpi) time point was collected by removing 200 μL from the apical and the basal compartment. After collection, medium was replaced apically and basally. Cultures were incubated for 72 h, with sample collection both apically and basally at 16, 24, 48 and 72 hpi. Collected medium samples were stored at −70 °C until further use.

#### 5.6.2 Viral infection with hCMV

Virus stocks of CMV10 were thawed in a water bath at 37 °C and centrifuged at 3.300 x g for 10 min at RT. Apical medium was removed from HIE and HIE+FIFs cultures and 100 µL virus stock supernatant was added to the apical side for 24 h at 37 °C, 5% CO2. After 24 h incubation, the virus containing medium was removed and inserts were washed three times with pre-warmed Advanced DMEM/F12. After washing, apical and basal medium was removed and collected for the 1 day post infection (dpi) time point. IntestiCult ODMh was added and medium was changed and collected every other day until 15 dpi. Collected medium samples were stored at −70 °C until further use.

#### 5.6.3 Median tissue culture infectious dose

Supernatant samples from EV-A71 and Echo 11 at 0 and 72 hpi were used to determine the presence of infectious viral particles; titrations were performed on the RD cell line. Seven ten-fold dilutions of each sample were performed and 50 µL of each dilution was added to a 96-well plate, after which 200 µL of specific cells were added. Plates were incubated for 7 days until scoring of the cytopathic effect (CPE) and calculation of the median tissue culture infectious dose (TCID50) according to the Reed and Muench Method (Reed and Muench, 1938). Values at 72 hpi were normalized to 0hpi to determine the increase of infectious particles.

#### 5.6.4 Viral RNA isolation and qPCR EV-A71 and Echo11

RNA was isolated from cultures infected with EV-A71 and E11. Media samples (25 μL) were added to 300 μL lysis buffer and the total RNA was isolated using the PureLink^TM^ RNA Mini kit (Thermo Fisher) according to the manufacturer’s instructions. Equal volumes of eluted RNA were used for reverse-transcription using the SuperScript™ II Reverse Transcriptase synthesis kit (Thermo Fisher). A volume of 5 μL of cDNA was used for quantitative PCR (qPCR) on a CFX Connect Real-Time PCR Detection System (Bio-Rad). SYBR Green qPCRs were performed using the pan entro primers 5 ’– GGC​CCT​GAA​TGC​GGC​TAA​T – 3’; reverse primer 5’ – GGG​ATT​GTC​ACC​ATA​AGC​C – 3’; as described previously (Benschop et al., 2010).

#### 5.6.5 Viral DNA isolation and qPCR CMV

Viral DNA was isolated from cultures infected with CMV. 100 µl media samples were collected from the apical and basal chamber and viral DNA was isolated using the ISOLATE II Genomic Kit (Meridian Bioscience,). The following primers and probe were used for quantitative PCR (qPCR) on a CFX Connect Real-Time PCR Detection System: forward primer 5 ’– CACGGTCCCGGTTTAGCA – 3’; reverse primer 5’ – CGT​AAC​GTG​GAC​CTG​ACG​TTT – 3’; and Taqman probe FAM–TGTAACCGCGATCCTCGGGCAGATA–TAMRA.

### 5.7 *In vitro* modelling of inflammation

To simulate an *in vivo* inflammatory environment and activate specific subsets of APCs, various combinations of cytokines were supplemented into the complete or intermediate intestinal mucosa models. ImmunoCult™ Dendritic Cell Maturation Supplement was added to IntestiCult ODMh in both the apical and basal chambers to induce DC activation. Alternatively, 10 ng/mL LPS and 50 ng/mL IFN-γ were supplemented to IntestiCult ODMh for classical macrophage activation (M1), or 10 ng/mL IL-4 for alternative macrophage activation (M2), also in the apical and basal chambers of inserts containing the complete intestinal mucosa model. For long-term experiments (over 48 h), medium changes were performed every other day with 300 µL and 700 µL in the apical and basal chambers, respectively. Each time, non-adherent APCs collected during these steps were rescued, and returned to the basal chamber.

### 5.8 Cell viability

#### 5.8.1 Quantification of intracellular adenosine triphosphate

Cell viability and cellular metabolic activity was determined based on intracellular adenosine triphosphate (ATP) production, using the 3D CellTiter-Glo Luminescent cell viability assay (Promega) as published previously (Wrzesinski et al., 2021) with minor modifications. Briefly, medium was aspirated and 100 µL PBS was added to each sample well of HIE. Cells were lysed with 100 µL 3D CellTiter-Glo lysis buffer and shaken in the dark for 30 min. Following incubation, the lysate was transferred to black clear bottom 96-well plates and the luminescence was measured in an H1 Synergy plate reader (BioTek). The data was normalized with reference to a standard curve for ATP (Sigma-Aldrich) and relevant controls.

#### 5.8.2 Extracellular lactate dehydrogenase

Cell toxicity was determined by extracellular lactate dehydrogenase (LDH) release, using the LDH-Glo™ Cytotoxicity assay (Promega) following manufacturer’s instructions. Briefly, culture medium samples (5 µL) were added to 95 µL LDH storage buffer and stored at −70 °C until further processing. The assay was performed by transferring 50 µL of the sample and LDH standard in LDH storage buffer to a black clear bottom 96-well plate. LDH detection reagent (50 µL) was added to standard and sample wells and incubated at room temperature in the dark for one hour. Luminescence was measured in an H1 Synergy plate reader. Data was normalized with reference to a standard curve of LDH and relevant controls.

#### 5.8.3 Quantification of live and dead cells by automated fluorescent cell count

Cell viability was assessed using Nucleocounter NC-250 (Chemometec), an automated fluorescent cell counter. Cell cultures were first dissociated into single-cell suspensions using either ACCUTASE or TrypLE. Collected cells were centrifuged at 300 x g for 5 min and resuspended in their respective culture medium. A 20 µL aliquot of the cell suspension was stained with 1 µL of AO-DAPI Solution 18 (Chemometec) to differentiate between live and dead cells. Samples were analysed using the Nucleocounter NC-250 to estimate cell viability.

### 5.9 Trans electrical epithelial resistance measurements (TEER)

TEER was measured using the EVOM2 or EVOM3 Epithelial Voltohmmeter (World Precision Instruments), routinely calibrated with a set of standard resistors. Resistance values of a coated empty insert were used as background. All TEER measurements were performed in triplicate per donor and the average was used for calculations.

### 5.10 FITC-dextran permeability assay

Paracellular permeability was assessed using the fluorescent tracer FITC-Dextran 4 kDa (FD4). A solution of 1 mg/mL FD4 was added to the apical chamber of cell culture inserts. After incubation on a shaker at 70 rpm and 37 °C for either 3 or 24 h, 100 µL aliquots of medium were sampled twice from the basal chamber and transferred to a black flat-bottom 96-well plate. Fluorescence intensity at 520 nm was measured using either a SpectraMax® M3 multi-mode microplate reader or a Synergy hybrid microplate reader, with the excitation wavelength set at 490 nm, a cut-off at 515 nm, and an emission wavelength at 520 nm. Data was normalised to a standard curve with known concentrations of FITC-Dextran in IntestiCult ODMh. The plate blank was established using a 100 µL aliquot of IntestiCult ODMh medium. Control measurements included a loading control (1 mg/mL FD4 in IntestiCult ODMh) and an empty insert control (cell culture insert without cells) for each experiment.

### 5.11 Gene expression analysis

Total RNA was purified using the RNeasy Mini kit spin columns or the Ambion PureLink® RNA Mini kit, according to the supplier’s protocol. RNA quantification was performed using Nanodrop 2000 (ThermoFisher Scientific, United States). Reverse transcription was carried out with High Capacity cDNA Reverse Transcription Kit with Complementary DNA (cDNA) samples diluted to a final concentration of 5 ng/ml. Quantitative real-time polymerase chain reaction (RT-qPCR) assay was performed with TaqMan™ Fast Universal PCR Master Mix (2X) no AmpErase™ UNG and 0.5 µl of primer/probe set. Predesigned qPCR assays were purchased from Integrated DNA Technologies (United States) and Applied Biosystems (United States) or were custom-designed using IDT PrimerQuest Tool (IDT, United States) and sequence homogeneity was confirmed by comparison to all available sequences on the GenBank database using BLAST (http://www.ncbi.nlm.nih.gov/BLAST/). The primers used in the study were purchased from IDT (Belgium) and are listed in Supplementary Table S1. RT-qPCR was performed using the StepOnePlus Real-Time PCR system (Life Technologies, United States). Standard curves and efficiency tests were generated for each target. Unless differently stated in the figure legends, gene expression levels are plotted as -ΔCt values, values are normalised to the average threshold cycle (Ct) values for the gene of interest (GOI) against the average Ct values for the reference gene (*TBP*), from 3 technical replicates.

### 5.12 Immunocytochemistry

Human foetal intestinal mucosa cultures were fixed in 4% (v/v) paraformaldehyde (PFA) or cold methanol for 30 and 10 min respectively at RT and stored in PBS at 4 °C. To reduce autofluorescence inserts were treated with 0.3% (w/v) Sudan Black B (Sigma- Aldrich, 199664-25G) in 70% (v/v) Ethanol for 30 min at RT. Inserts were immersed and washed five times in PBS. Overnight blocking was performed, using Fish Serum Blocking Buffer (ThermoFisher, 37527) at 4 °C. A Liquid Blocker Super PAP Pen (Daido Sangyo) was used to circle two parts of a microscopy slide (VWR, 631-1161) and membranes were cut out form the inserts, using a scalpel, and placed within the PAP circle. Overnight primary antibody incubation at 4 °C was performed, in a humidified chamber. After incubation, membranes were washed three times with Tris-buffered saline (TBS)-Tween (150 mM NaCl, 50 mM Tris-HCL buffer and 1% w/v Tween20) (TBS; EMD Millipore 524750, Tween20; Sigma-Aldrich) followed by secondary antibody and Hoechst 33342 (Invitrogen, H3570, 1:1000 in Fish Serum Blocking Buffer) incubation for 2 h at RT. After three washes with PBS, membranes were mounted using ProLong TM Glass Antifade Mountant (ThermoFisher Scientific, P36984). A EVOS TM M5000 or Leica TCS SP8-X microscope with HC Plan Apochromat 40x and 63x oil objective were used to image the slides. Leica LAS X Software (Leica Microsystems) was used for analysis.

Human adult intestinal mucosa cultures were fixed in 4% (v/v) paraformaldehyde (PFA) or cold methanol for 30 and 10 min respectively at RT and stored in PBS at 4 °C. According to the target localisation, cells were permeabilised with 0.2% Triton-X 100 in PBS for 10 min at room temperature. Treatment with 1% BSA in PBS for 60 min was used to block non-specific binding. Subsequently, cultures were treated with primary antibodies at specific dilutions for each target (ranging from 1:50 to 1:400) for 60 min at RT. Cultures were then incubated with secondary antibodies (dilution 1:400) in the dark for 60 min and counterstained with 4′,6-diamidino-2-phenylindole (DAPI) for 1 min. Lastly, cultures were left with 0.5 ml PBS for observation with Leica DMi8 inverted fluorescence microscope (Leica Microsystems, Germany). Mean fluorescent intensity per image was quantified using ImageJ software (NIH, United States), and normalised to the number of cells per image indicated by DAPI stain. A list of the antibodies used in this study can be found in Supplementary Table S2.

### 5.13 Flow cytometry analysis

Cells were enzymatically dissociated with TrypLE Express for 15 min at RT. The collected cell suspension was then mechanically dissociated followed by the addition of 2 mL FACS buffer (PBS supplemented with 0.1% BSA) was added. Cells were centrifuged at 300 x g for 5 min at 4 °C, and the supernatant was discarded. The pellet was resuspended in 1 mL FACS buffer, and the cells counted with an automatic cell counter. For each flow cytometry assay, 250,000 live cells were distributed in each sample tube. If epitope localisation was intracellular, cells were fixed in 4% PFA or cold methanol for 10 min at RT, followed by permeabilised with 0.2% Triton-X 100 in PBS for 10 min at RT. Blocking was performed using Human TruStain FcX™, Fc Receptor Blocking Solution for 10 min at RT. Then, cells were stained with conjugated antibody cocktails diluted in 100 µL FACS buffer for 45 min in the dark at RT. Unless otherwise stated, DAPI was used as a viability stain for 1 min at RT. Following staining samples were washed three times in 2 mL FACS buffer followed by centrifugation at 300 x g for 5 min. Samples were then resuspended in 100µL FACS buffer and added to a 96-well plate. At least 10,000 cells were analysed using CytoFlex (Beckman Coulter, United States) together with CytExpert Software (Beckman Coulter, United States). Compensation matrices were generated for each multi-colour panel of antibodies, with the following gating strategy. The stability of each run was assessed by looking at the forward scatter area (FSC-A) in time. Eventual irregularities in the flow were gated out from the analysis. A doublet exclusion gate was also set on an FSC-A against forward scatter height (FSC-H) plot. Dead cells were eliminated from the analysis by including a live/dead stain in each assay. The remaining events represented the population of interest. A list of the conjugated antibodies used in this study can be found in Supplementary Table S3.

### 5.14 Statistical analysis

Unless otherwise specified, all experiments were conducted in triplicates, and data are expressed as mean ± standard deviation. Statistical analysis was conducted using GraphPad Prism 8 software (GraphPad Software Inc., United States). Normality test (D'Agostino-Pearson normality test) and equality of variances tests (Bonett’s test and Levene’s test) were conducted. When the assumptions of parametric analysis were confirmed, a student’s t-test was used to compare two groups, and a one-way analysis of variance (ANOVA) was used to compare more than 2 groups, followed by Bonferroni’s post-hoc test. Where more than one factor influenced the variable being measured, two-way ANOVA and Tukey’s multiple comparison tests were used to assess for a significant effect of each factor, as well as an interaction between factors. When the assumptions of parametric analysis were violated, a Mann-Whitney U test or a Kruskal-Wallis test were used to compare two or more independent groups, respectively. Statistical significance levels were denoted by asterisks, adhering to the NEJM style [p > 0.05 = (ns); p < 0.05 (*); p < 0.01 (**); p < 0.001 (***)].

## Data Availability

The datasets presented in this study can be found in online repositories. The names of the repository/repositories and accession number(s) can be found below: https://doi.org/10.6084/m9.figshare.32091250.
