# Supplementary material for: A human intestinal epithelial-mesenchyme-immune triple culture system for disease modelling
Source: Front Cell Dev Biol. 2026 Jul 7;14:1867636. doi: 10.3389/fcell.2026.1867636 (PMC13385409; doi:10.3389/fcell.2026.1867636)
Supplement: Supplementary file 1 [file Supplementaryfile1.docx]

# Supplementary Information

## Supplementary Tables

### Supplementary Table 1 - List of qPCR predesigned assays used in gene expression analysis.

| **Gene Name** | **Gene Symbol** | **IDT Cat #** |
| --- | --- | --- |
| Actin Alpha-2, Smooth Muscle | *ACTA2* | Hs.PT.56a.2542642 |
| Cluster of Differentiation 14 | *CD14* | Hs.PT.56a.3118607.g |
| Cluster of Differentiation 163 | *CD163* | Hs.PT.58.3564170 |
| Mannose Receptor C Type 1 (MRC1) | *CD206* | Hs.PT.58.15093573 |
| Dendritic Cell-Specific Intercellular Adhesion Molecule-3-Grabbing Non-Integrin (DC-SIGN) | *CD209* | Hs.PT.58.40094997 |
| Cluster of Differentiation 80 | *CD80* | Hs.PT.56a.38577902 |
| Cluster of Differentiation 83 | *CD83* | Hs.PT.58.41034901 |
| Cluster of Differentiation 86 | *CD86* | Hs.PT.58.21526437 |
| Collagen Type I Alpha 1 | *COL1A1* | Hs.PT.58.15517795 |
| Collagen Type III Alpha 1 | *COL3A1* | Hs.PT.58.4249241 |
| Collagen Type IV Alpha 1 | *COL4A1* | Hs.PT.58.15679435 |
| Fibronectin | *FN1* | Hs.PT.58.40005963 |
| GLI Family Zinc Finger 1 | *GLI1* | Hs.PT.58.26486279 |
| Gremlin 1 | *GREM1* | Hs.PT.58.21084086 |
| Gremlin 2 | *GREM2* | Hs.PT.58.38639879 |
| Interleukin 12 Subunit Beta | *IL12B* | Hs.PT.58.2925830 |
| Interleukin 1 Beta | *IL1B* | Hs.PT.58.1518186 |
| Interleukin 33 | *IL33* | Hs.PT.58.28311718 |
| Interleukin 6 | *IL6* | Hs.PT.58.40226675 |
| Interleukin 8 (CXCL8) | *IL8* | Hs.PT.58.39926886.g |
| Noggin | *NOG* | Hs.PT.58.27300029.g |
| Platelet Derived Growth Factor Receptor Alpha | *PDGFRA* | Hs.PT.58.45699973 |
| Podoplanin | *PDPN* | Hs.PT.58.40724642 |
| 18S Ribosomal RNA | *RNA18S5* | Hs.PT.39a.22214856.g |
| R-Spondin 1 | *RSPO1* | Hs.PT.58.4953117 |
| R-Spondin 3 | *RSPO3* | Hs.PT.58.2082266 |
| Tata-Box Binding Protein | *TBP* | Hs.PT.39a.22214825 |
| Cluster of Differentiation 90 (CD90) | *THY1* | Hs.PT.58.22816234 |
| Tenascin C | *TNC* | Hs.PT.58.2529606 |
| Tumor Necrosis Factor | *TNF* | Hs.PT.58.45380900 |
| Vimentin | *VIM* | Hs.PT.58.38906895 |
| Wnt Family Member 2B | *WNT2B* | Hs.PT.58.26560263 |
| Wnt Family Member 5A | *WNT5A* | Hs.PT.58.22221435 |

### Supplementary Table 2: List of antibodies used for immunocytochemistry analysis.

| **Description** | **Cat#** | **Supplier** |
| --- | --- | --- |
| Anti-Collagen I antibody | ab90395 | Abcam |
| Anti-Collagen III antibody | ab7778 | Abcam |
| Anti-Collagen IV antibody | ab6586 | Abcam |
| Anti-Fibronectin antibody | ab2413 | Abcam |
| Anti-PDGFR alpha antibody [EPR22059-270] | ab203491 | Abcam |
| Anti-RSPO3 antibody | ab233113 | Abcam |
| Anti-α smooth muscle actin antibody | ab5694 | Abcam |
| APC anti-human CD103 | 350216 | Biolegend |
| EPCAM | AF960 | R&D Systems |
| FITC anti-human CD90 | 328107 | Biolegend |
| GLI1 | MA5-38530 | Invitrogen |
| Goat anti-Mouse IgG1, Alexa Fluor 647 | A-21240 | Invitrogen |
| Goat anti-Rabbit IgG, Alexa Fluor 488 | A-11034 | Invitrogen |
| IE1 |  | Gifted by Prof. Dr. William Britt, The University of Alabama |
| Lysozyme EC 3.2.1.17 | A0099 | Dako |
| PE anti-human CD108 | 376604 | Biolegend |
| Recombinant Anti-Podoplanin /  gp36 antibody [EPR7072] | ab128994 | Abcam |
| RSPO1 | PA5-121183 | Invitrogen |
| Anti-Human CD45 Antibody, Clone HI30  (100 tests) | 60018AZ | STEMCELL Technologies |
| ZO-1 Monoclonal Antibody (ZO1-1A12),  Alexa Fluor 488 | 339188 | Themo Fisher Scientific |

### Supplementary Table 3 - List of antibodies used for flow cytometry analysis.

| **Description** | **Cat#** | **Supplier** |
| --- | --- | --- |
| Anti-DC-SIGN antibody [UW60.1] (APC) | ab180548 | Abcam |
| Anti-Human CD14 Antibody, Clone M5E2 | 60004PE | STEMCELL Technologies |
| APC anti-human CD103 | 350216 | Biolegend |
| APC anti-human CD83 | 305312 | Biolegend |
| FITC anti-human CD86 | 374204 | Biolegend |
| PE anti-human CD108 | 376604 | Biolegend |
| PE anti-human CD45 | 304007 | Biolegend |
| PE anti-human CD80 | 375410 | Biolegend |
| Recombinant Anti-Mannose Receptor antibody [EPR6828(B)] (Alexa Fluor® 488) | ab195191 | Abcam |
| Anti-Human CD45 Antibody, Clone HI30 (100 tests) | 60018AZ | STEMCELL Technologies |

###

### Supplementary Table 4 - Marker genes defining cell clusters identified by scRNA-seq.

| **Cell cluster** | **Key markers** | **References** |
| --- | --- | --- |
| Differentiated epithelium & Epithelial cells 1 (Maturing epithelium) | *MEP1A, MTTP, APOBEC1, CYP3A4, FAPB1, FAPB2, ANPEP, PHGR1, ZG16, REG4* | ^32,74,79,100,101^ |
| Epithelial cells 2 (Secretory cells) | *ANKRD36C, FER1L6, MUC4, MUC5B* | ^101,102^ |
| Epithelial cells 3 (Immature epithelium) | *OMFL4^high^*, *GPX2, DMBT1, PDZK1IP1, LCN2, UBD, NOS2, SERPINA1, CD74* | ^74,100,101,103,104^ |
| Epithelial cells 4 (Activated epithelial cells) | *UBD, LTB, IL23A*, *TNF*, *ITGB8,* *CXCL1*, *CX3CL1* | ^105-107^ |
| Cycling cells | *MKI67, TOP2A, RGMB, CDK1, PBK, RRM2, PCNA* | ^74,100,101^ |
| Intestinal fibroblasts | *VIM, PDGFRA, FOXL1, PDPN, COL1A1, COL1A2, COL3A1, COL4A1 TNC, THY1, ACTA2, WNT5A, BMP1, BMP2, GREM2* | ^74,101,108,109^ |

**Supplementary Table 5** – Donor and replicate configurations utilised in this study

| Model | Strengths | Limitations | References |
| --- | --- | --- | --- |
| HIO 3D | - Self organising 3D structure - Scalability - Easy to employ - Isogenic model - Commercially available culture media | - No access to luminal compartment unless invertedly polarised - Lack of additional cell types aside from epithelium - No barrier function analyses possible | - ^110,111^ |
| IEC monolayer | - Barrier creating apical and basolateral compartment - Easy to employ - Isogenic model - Commercially available culture media | - Lack of non-epithelial cell types - Need for abluminal inoculation for some viruses - Limited culture longevity | - ^47,72,73^ |
| iPSC-derived intestinal mucosa model | - Increased physiological relevance - Use for complex pathogen-interaction studies - Culture longevity - Isogenic model | - Increased culture pipeline complexity - No fibroblast or immune cell compartment - Generally a more foetal-like | - ^75,112-114^ |
| Intestinal model on microfluidic chip | - Physiological flow - Permits co-culture with microbiome | - Specialised technical expertise and equipment necessary - Microfluidic platform is not easily scalable - No immune compartment co-culture published | - ^75,115^ |

**Supplementary Table 6** – Donor and replicate configurations utilised in this study

| Figure | Donor | Biological N | Technical N |
| --- | --- | --- | --- |
| 2C | Adult | 3 (IFs) 5 (IECs) | 2 |
| 2D | Adult | 2 | 31 |
| 2E | Adult | 3 (IFs) 5 (IECs) | 2 |
| 2F | Adult | 2 (IECs+IF, IFs) 1 (IECs) | 2 (IECs+IF) 3 (IECs) 4 (IFs) |
| 3A | Adult | 3 (IFs) 5 (IECs) | 2 |
| 3B | Adult | 4 (IECs, IFs) 8 (IECs) | 1 |
| 3E | Adult | 8 | 2 |
| 3F | Adult | 6 (DCs) 8 (DCs+IECs+IFs) 4 (DCs+IECs) | 1 |
| 4 | Foetal/Adult | 2 (28,528 cells) | 6 |
| 5B | Adult | 8 | 2 |
| 5D | Adult | 8 | 1 |
| 5E | Adult | 8 | 1 |
| 6B | Foetal | 2 | 3 |
| 6C | Foetal | 2 | 3 |
| 6D | Foetal | 2 | 2 |
| 6E | Foetal | 2 | 2 |
| Sup figure 1A | Adult | 4 | 2 |
| Sup figure 1C | Adult | 4 | 2 |
| Sup figure 1D | Adult | 2 | 31 |
| Sup figure 1E | Adult | 1 (IECs) 2 (IECs+IFs) | 3 (IECs) 4 (IECs+IFs) |
| Sup figure 1G | Adult | 2 | 35 |
| Sup figure 2A | Foetal/Adult | 2 (a-IECs) 3 (a-IFs) 4 (f-IECs, f-IFs) | 2 |
| Sup figure 2C | Foetal/Adult | 3 (a-IFs) 4 (f-IFs) | 2 |
| Sup figure 3B | Foetal/Adult | 2 | 3 |
| Sup figure 4B | Adult | 1 | 6 |
| Sup figure 4C | Adult | 4 | 1 |
| Sup figure 4D | Adult | 4 | 1 |
| Sup figure 4E | Adult | 4 | 1 |
| Sup figure 5 | Foetal/Adult | 2 | 6 |
| Sup figure 6C | Adult | 2 | 4 |
| Sup figure 6D | Adult | 3 | 1 |
| Sup figure 7B | Foetal | 2 | 2 |
| Sup figure 8A-D | Foetal | 2 | 3 |
| Sup figure 8E | Foetal | 2 | 2 |
| Sup figure 8F | Foetal | 2 | 2 |

## Supplementary Figures

**
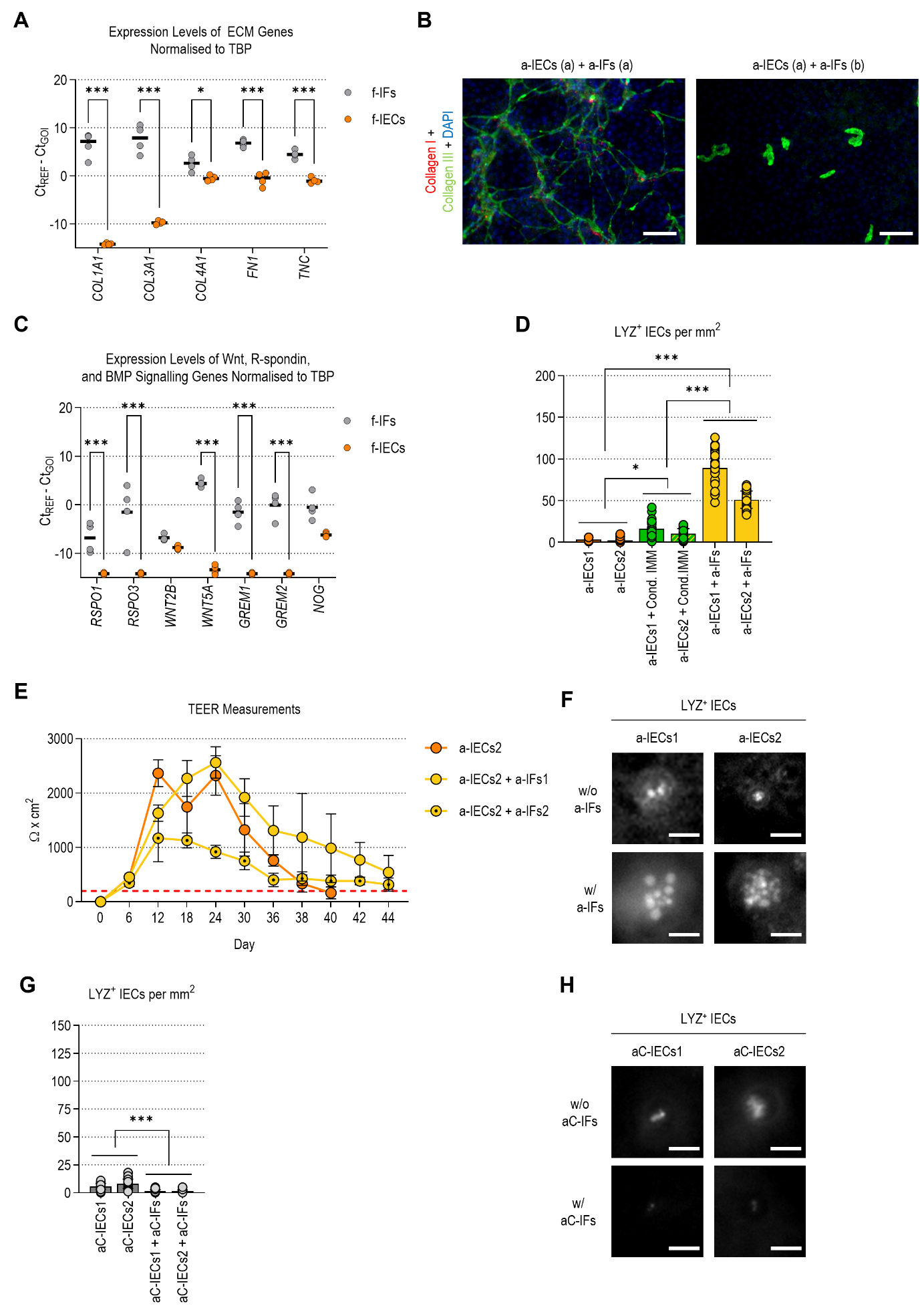
**

**Supplementary Figure 1 – Additional data on the epithelial-mesenchymal crosstalk. (A)** RT-qPCR analysis of ECM genes performed on f-IECs and f-IF mono-cultures maintained for 14 days on cell culture inserts in IMM. Gene expression levels are plotted as -ΔCt values. Each point represents a biological replicate (n = 4), grey and orange colours highlight data related to IFs and IECs, respectively. Gene names are listed at the bottom of the plot. Statistical analysis employed a two-way ANOVA, along with Šídák’s multiple comparison test. **(B)** Representative immunofluorescence images of collagen type I and collagen type III expression at day 14 in IEC-IF co-cultures seeded either together on the same side [IECs (a) + IFs (a)] or on opposite sides of the insert membrane [IECs (a) + IFs (b)] (scale bars = 100 µm). **(C)** RT-qPCR analysis of Wnt, R-spondin, and BMP signalling genes performed on f-IECs and f-IFs cultures maintained for 14 days on cell culture inserts in the same medium. Gene expression levels are plotted as -ΔCt values. Each point represents a biological replicate (n = 4), grey and orange colours highlight data related to IFs and IECs, respectively. Gene names are listed at the bottom of the plot. Statistical analysis employed a two-way ANOVA, along with Šídák’s multiple comparison test. **(D)** Bar graph summarising the results of the Paneth cells quantification based on LYZ immunostaining. The bars represent the mean and standard deviation (biological n = 2). Statistical analysis employed a one-way ANOVA, along with Tukey’s multiple comparison test. **(E)** TEER measurements performed on additional IECs, and IEC-IF co-cultures maintained in IMM. TEER measurements were normalised to the surface area of the cell culture inserts (0.332 cm^2^) and plotted as Ω·cm^2^ (y-axis) over time (x-axis). Each point represents the mean of two biological replicates at, and the bars indicate the standard deviation of these measurements. Orange, and yellow colours represent data pertaining to IECs and IEC-IF co-cultures. Dotted circle and hexagon symbols denote data related to IEC-IF co-cultures incorporating IFs sourced from different donors. **(F)** Representative immunofluorescence images of LYZ expression in Paneth cells at day 14 in a-IECs and a-IECs+a-IFs maintained in IMM (scale bars = 10 µm). Adult IECs derived from different donors are indicated with distinct numerical suffixes (e.g. a-IECs1 and a-IECs2). **(G)** Bar graph summarising the results of the Paneth cells quantification on adult colon IECs (aC-IECs) and adult colon IEC+IF co-cultures (aC-IECs+aC-IFs), based on LYZ immunostaining (biological n = 2). The bars represent the mean and standard deviation. Statistical analysis employed a one-way ANOVA, along with Tukey’s multiple comparison test. **(H)** Representative immunofluorescence images of LYZ expression at day 14 in aC-IECs, and aC-IEC+aC-IF co-cultures maintained in IMM (scale bars = 10 µm).

**
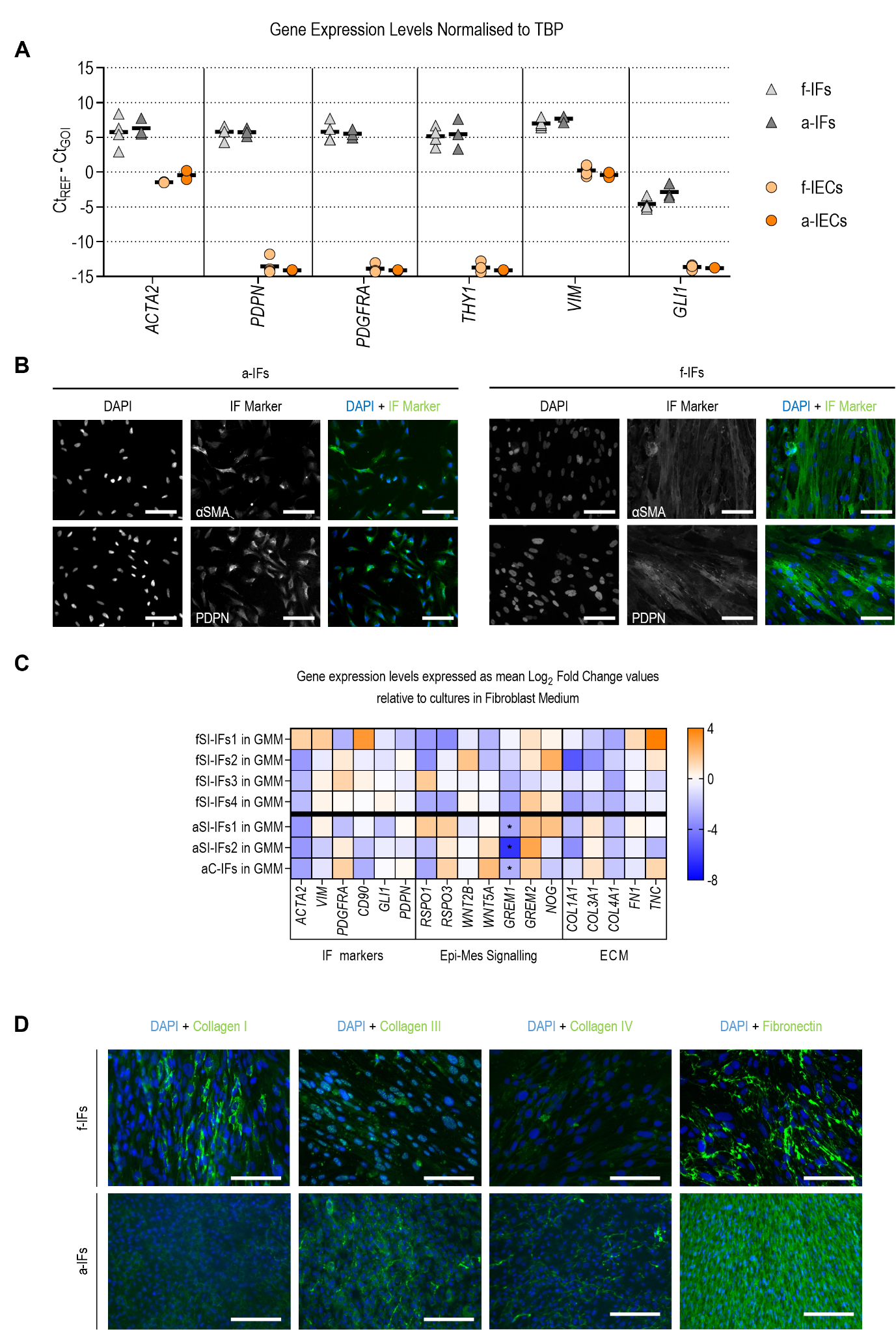
**

**Supplementary Figure 2 - IFs expressed markers characteristic of their identity, and maintained their phenotypical and functional features in IMM. (A)** Gene expression analysis by RT-qPCR on IFs cultured for 14 days in cell culture inserts in IMM. Gene expression levels are plotted as -ΔCt values. Each point represents a biological replicate (n = 4), triangle and circle symbols indicate data relative to IFs (orange) and IECs (grey), respectively, while light and dark colours highlight data related to foetal and adult cells, respectively. Gene names are listed on x-axis. **(B)** Representative immunofluorescence images of αSMA and PDPN expression in adult and foetal IFs cultured for 14 days in cell culture inserts in IMM (scale bars = 100 µm). For both adult and foetal IFs, the panel of three images shows from left to right: (i) DAPI staining the cell nuclei; (ii) the protein of interest; and (iii) the overlay of these two channels with blue and green highlighting the DAPI and target protein, respectively. **(C)** Heatmap representing the Log_2_ fold-change (Log_2_FC) values in gene expression of adult and foetal IF cultures maintained for 14 days on cell culture inserts in IMM, compared to their standard culture conditions in fibroblast medium (biological n = 3). Blue and orange colours indicate downregulation and upregulation of gene expression, respectively. Gene names are listed at the bottom of the heat map and are clustered by categories, related to IFs markers, epithelial-mesenchymal cell signalling, and ECM components. **(D)** Representative immunofluorescence images of collagen type I, collagen type III, collagen type IV, and fibronectin expression in adult and foetal IFs cultures maintained for 14 days on cell culture inserts in IMM (scale bars = 100 µm).


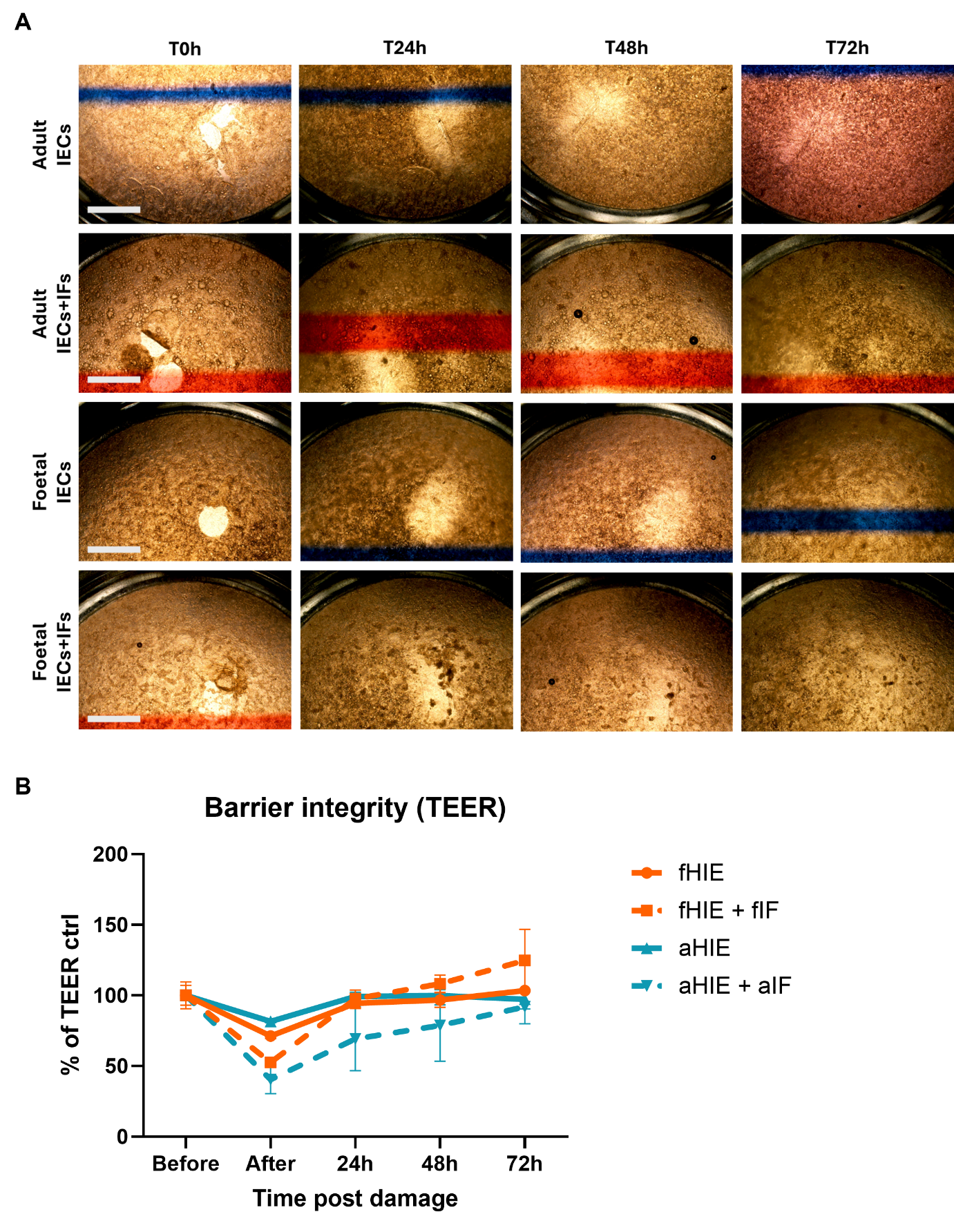


**Supplementary Figure 3** - **The regenerative capabilities of IEC and IEC+IF cultures**. **(A)** Brightfield images taken at 5x magnification every 24 hours until 72 hours after inflicted damage to the epithelial layer of adult/ foetal IECs and IECs + IFs cultures. Scale bar = 1mm. **(B)** TEER values of foetal and adult IECs and IECs + IFs cultures before inflicted damage, following the injury until 72 hours after infliction (biological n = 2, technical n = 3). TEER values were normalised to and depicted as percentage of undamaged control cultures. Standard deviation is plotted as error bars.

**
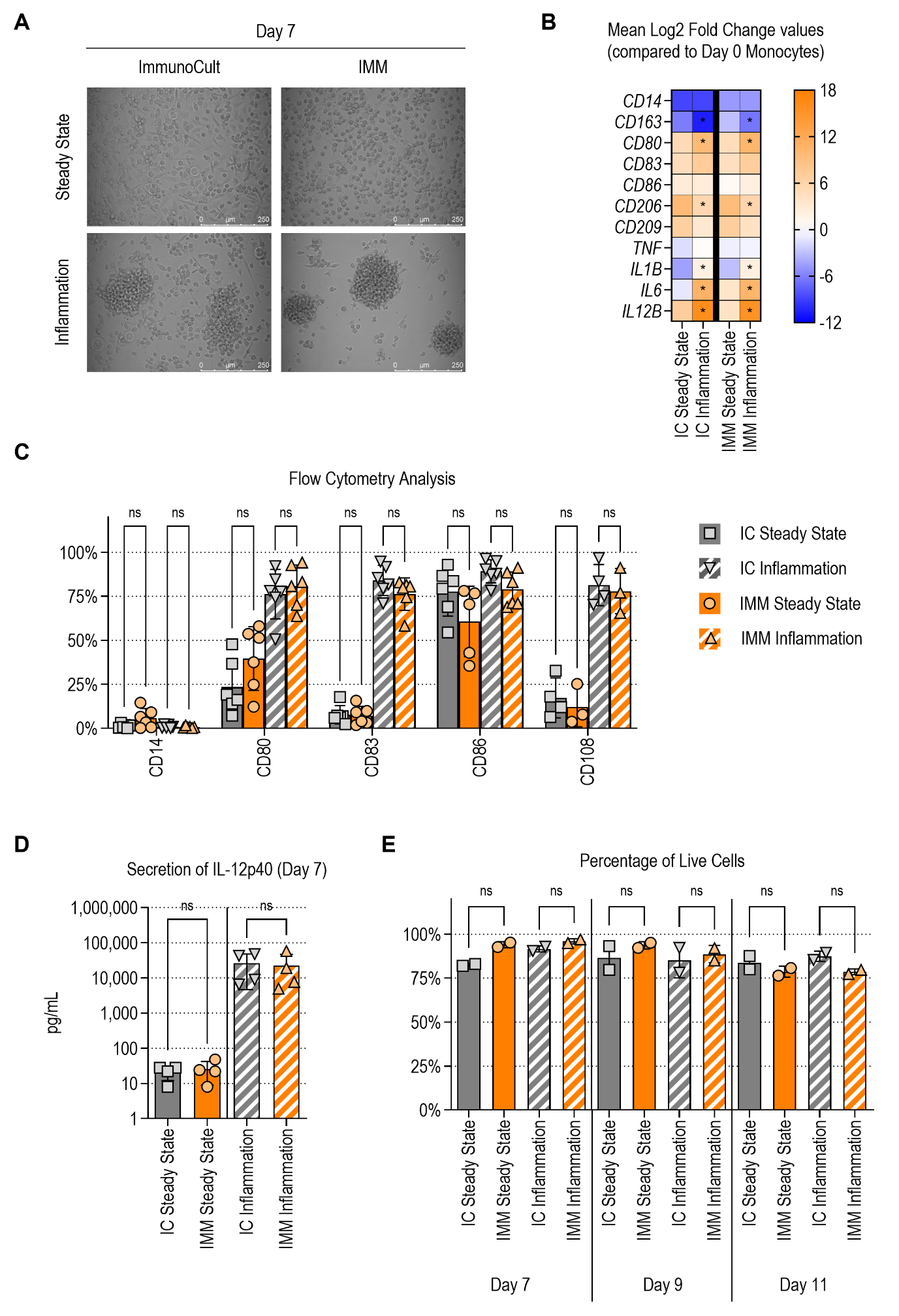
**

**Supplementary Figure 4 - DCs retain phenotype and functions in IMM. (A)** Representative phase contrast images of DCs cultured in ImmunoCult™ DC Differentiation Medium (IC) or intestinal mucosa medium (IMM) (scale bars = 250 µm). **(B)** Analysis of gene expression by qPCR in DCs at day 7 cultured in different media formulations. All samples were compared to monocyte progenitors to calculate the relative gene expression (mean + SD, technical n = 6) and expressed as Log2 fold changes. Orange colour indicates upregulation in gene expression (Log2 fold change > 0), blue colour indicates downregulation (Log2 fold change < 0). **(C)** Flow cytometry analysis of CD14, CD80, CD83, CD86, and CD108 protein levels at day 7 in DCs cultured in either IC or IMM, comparing steady state and inflammation conditions. Each dot represents a biological replicate (n = 6). The bars represent the mean and the standard deviation. **(D)** Dot plot indicating the concentration of IL-12p40 secreted by DCs cultured in either IC or IMM, comparing steady state and inflammation conditions. Each dot indicates a biological replicate (n = 4) and the horizontal bars represent the mean. **(E)** Cell viability assessed at day 7, 9 and 11 by AO-DAPI stain in DCs cultured in either IC or IMM, comparing steady state and inflammation conditions. Each dot represents a biological replicate (n = 2) and the horizontal bars represent the mean. Media conditions analysed are listed below.


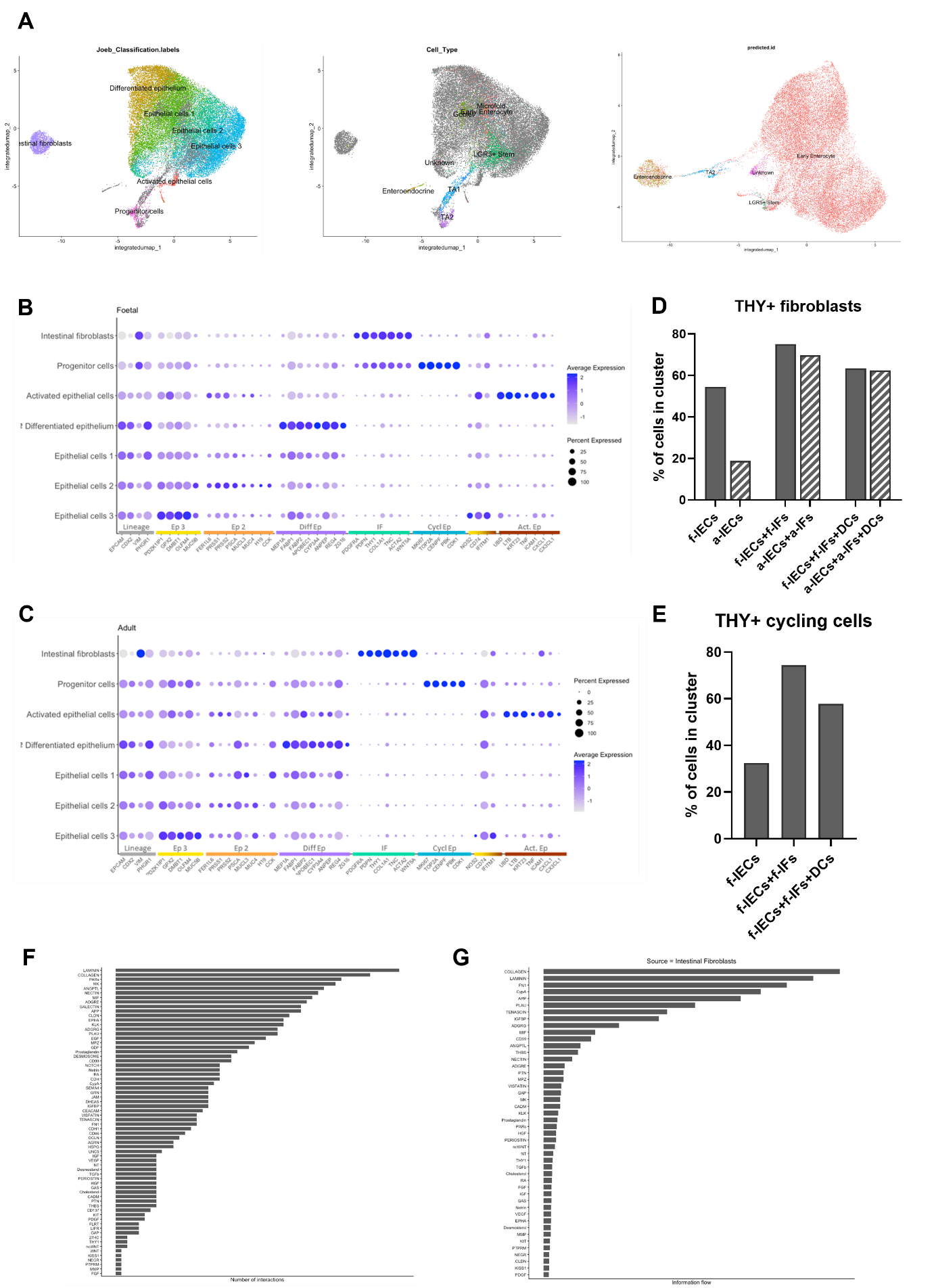


**Supplementary Figure 5 – Single cell RNA sequencing analysis of gene expression between foetal and adult cultures with IECs, IFs and DCs. (A)** UMAPs of our data integrated with GSE119969 dataset. Dot plot of defining markers for each cell cluster in the foetal **(B)** and adult **(C)** dataset (biological n = 2, technical n = 6). Values are scaled with 2 (blue/red) representing the highest, normalised, expression of a given gene and 0 (white) representing the lowest normalised expression. Dot size indicates the percentage of total cells in a cluster that express the gene of interest. *THY1* expressing cells within the **(D)** intestinal fibroblast and **(E)** progenitor cluster of foetal and adult IEC, IEC + IF and IEC cultures after 15 days of culture. **(F)** Signalling pathways ranked by number of significant ligand-receptor interactions from all cell types. **(G)** Signalling pathways ranked by number of significant ligand-receptor interactions from intestinal fibroblast cell type only.


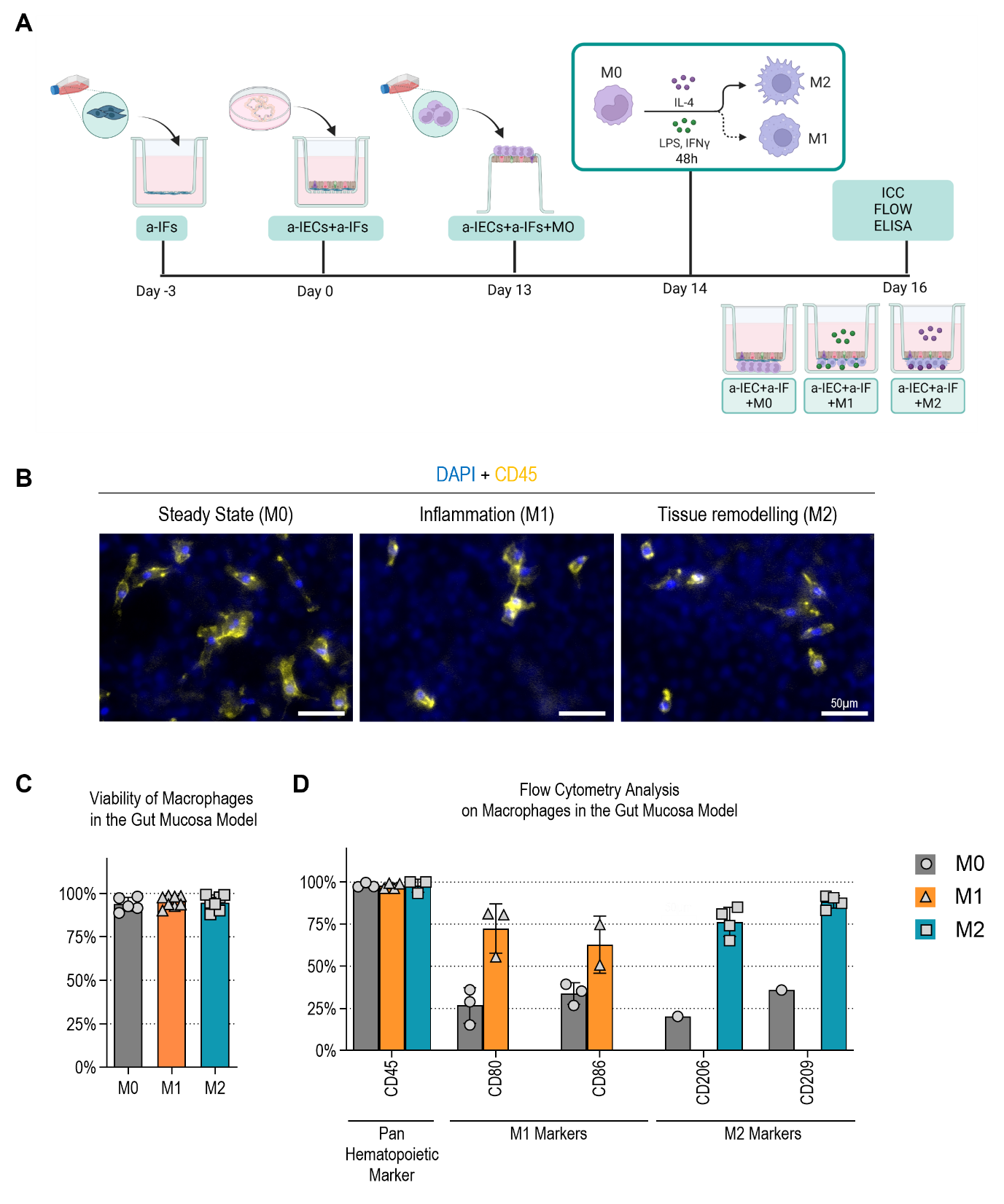


**Supplementary Figure 6 - Macrophages in the intestinal mucosa model maintain viability, phenotype, and functionality. (A)** Schematic representation of the workflow for modelling inflammation in the intestinal mucosa using the triple-culture model with MΦs. **(B)** Representative immunofluorescence images at day 14 displaying DAPI and CD45 expression in MΦs attached to the basal side of the inserts containing the intestinal mucosa triple-culture, maintained in IMM, either with or without supplementation of M1 or M2 activation factors (scale bars = 50 µm). **(C)** Cell viability assay based on acridin orange-DAPI stain performed at day 14 on MΦs located in the basal chamber of the intestinal mucosa model maintained in IMM, either with or without supplementation of M1 or M2 activation factors (10 ng/mL LPS + 50 ng/mL IFN-γ, or 10 ng/mL IL-4 respectively). Data points on the graph represent data related to 2 biological replicates and 4 technical replicates, with bars representing the mean and standard deviation. **(D)** Flow cytometry analysis of CD45, CD80, CD86, CD206, and CD209 expression levels at day 14 in M0, M1, or M2 macrophages cultured in the intestinal mucosa model in IMM. Each point represents data related to MΦ in a specific intestinal mucosa model (biological n = 3). The bars represent the mean with the standard deviation. Full grey bars with square symbols indicate data related to M0 macrophages, whilst striped bars with triangle symbols indicate data relative to M1 macrophages, and chequered bars with circle symbols indicate data relative to M2 macrophages.


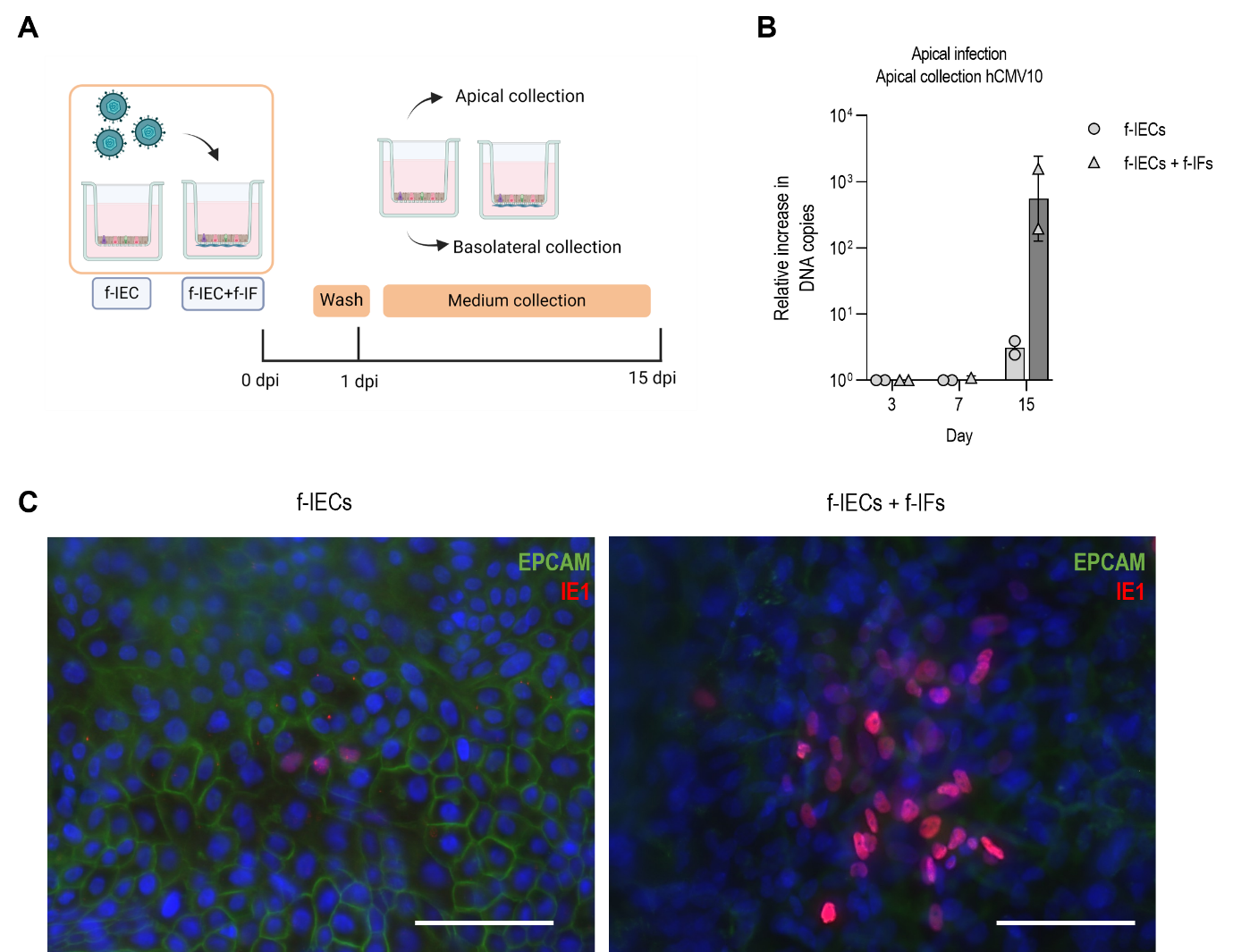


**Supplementary Figure 7 - hCMV infection of IEC and IEC-IF cultures. (A)** Schematic representation of viral inoculation and readouts. **(B)** Average relative increase in hCMV10 DNA copy numbers over time (biological n = 2, technical n = 2, error bars = SD). f-IEC monocultures and f-IECs+f-IFs are represented by circular and triangular grey symbols, respectively. **(C)** Immunofluorescence images at day 15 displaying the epithelial marker EPCAM in green and immediate early antigen (IE1) in red (scale bars = 100 µm).


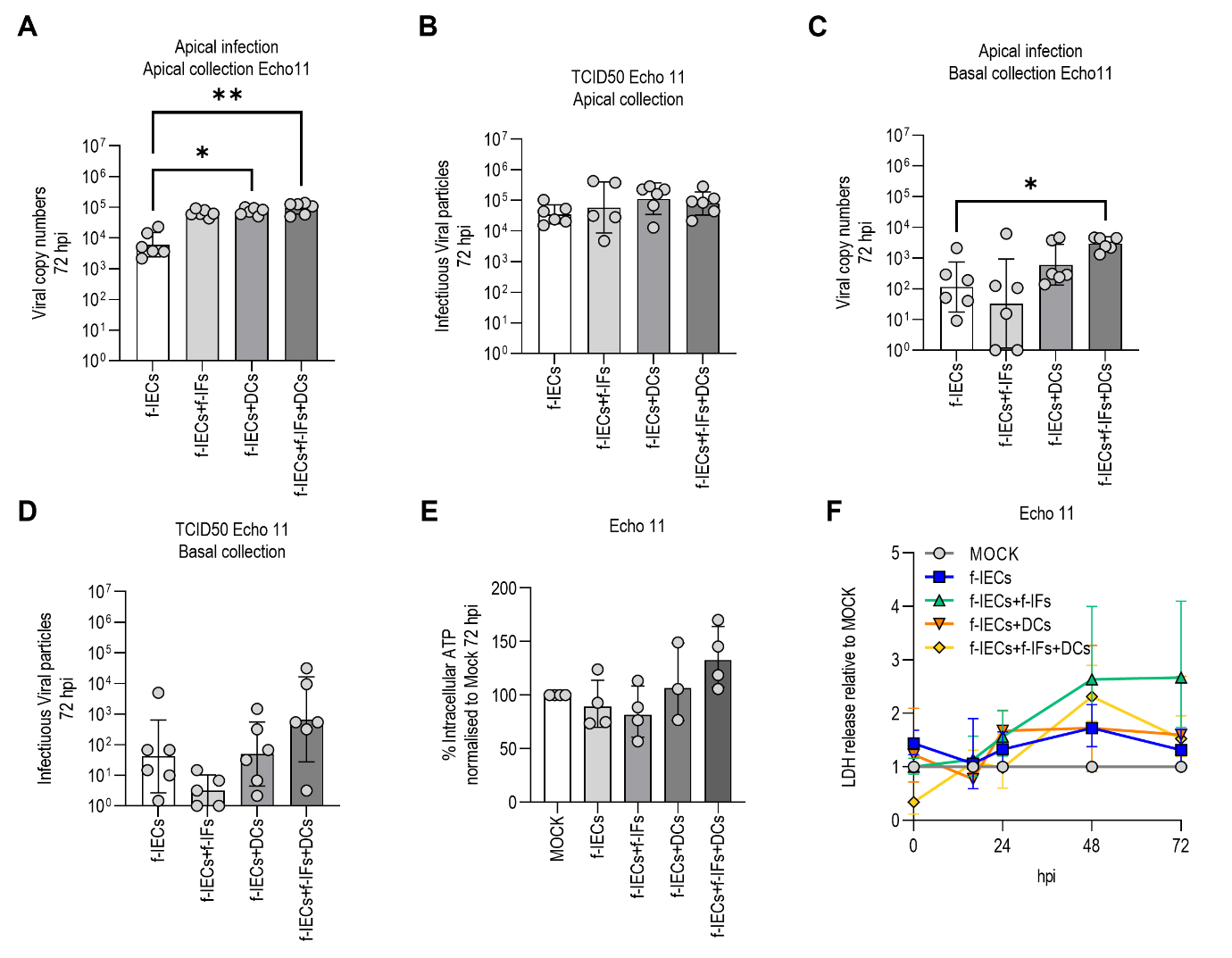


**Supplementary Figure 8 - EV-A71 and Echo 11 infection in a foetal mono-cultures, co-cultures and intestinal mucosa model. (A)** Average viral copy numbers in apical-collected medium following apical inoculation of Echo11, 72 hours post-infection (biological n = 2, technical n = 3, error bars = SD). **(B)** Average viral copy numbers in basal-collected medium following apical inoculation of Echo11, 72 hours post-infection (biological n = 2, technical n = 3, error bars = SD). **(C)** Average infectious viral particles in apical collected medium following apical inoculation of Echo11, 72 hours post-infection (biological n = 2, technical n = 3, error bars = SD). **(D)** Average infectious viral particles in basal-collected medium following apical inoculation of Echo11, 72 hours post-infection (biological n = 2, technical n = 3, error bars = SD). **(E)** Average increase (%) of intracellular ATP indicative of metabolic activity (biological n = 2, technical n = 2, error bars = SD) **(F)** LDH release relative to the uninfected mock condition for all models over time (biological n = 2, technical n = 2, error bars = SD). Statistical analysis employed a Kruskal-Wallis along with Dunn multiple comparison tests.


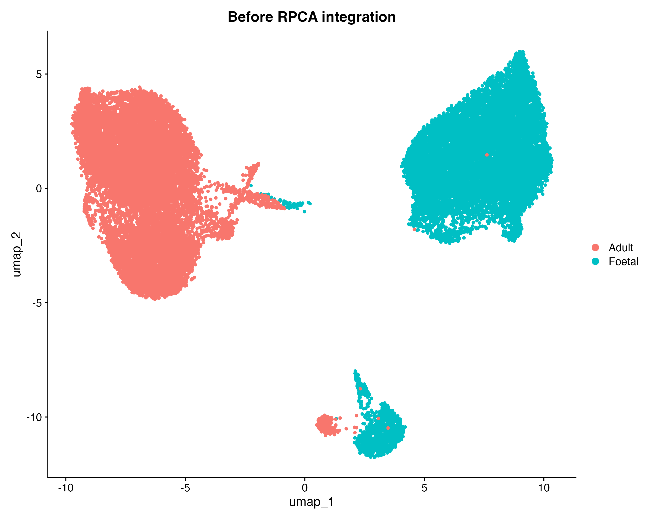

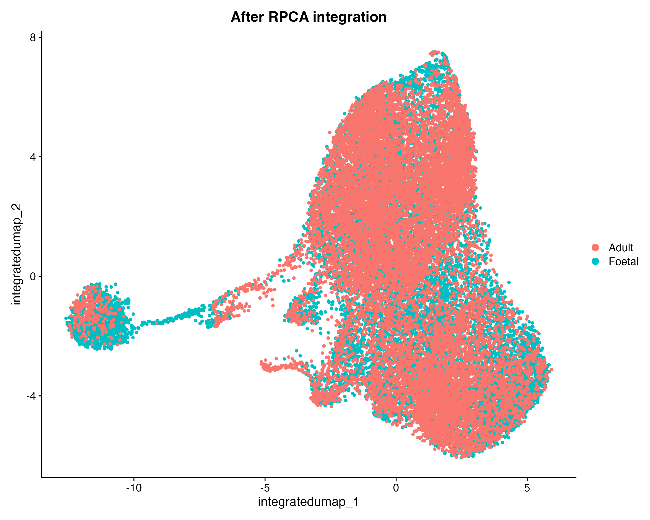


**Supplementary Figure 9 - UMAP before and after RPCA Integration.** To correct for donor-specific effects between the adult and foetal samples, we integrated the datasets using the Reciprocal PCA (RPCA) workflow implemented in Seurat v5. Each sample was first processed independently using normalization, variable feature selection, and PCA. Prior to integration, a joint UMAP embedding showed strong donor separation, indicating substantial batch effects. Integration was performed using the Reciprocal PCA integration method implemented in Seurat’s *IntegrateLayers* function. RPCA was chosen because it conservatively aligns shared biological structure while avoiding over-correction when samples contain partially overlapping cell types. After integration, PCA and UMAP were recomputed, and the embedding showed substantial mixing of adult and foetal cells, demonstrating that donor-driven variation was effectively removed while preserving biological heterogeneity.


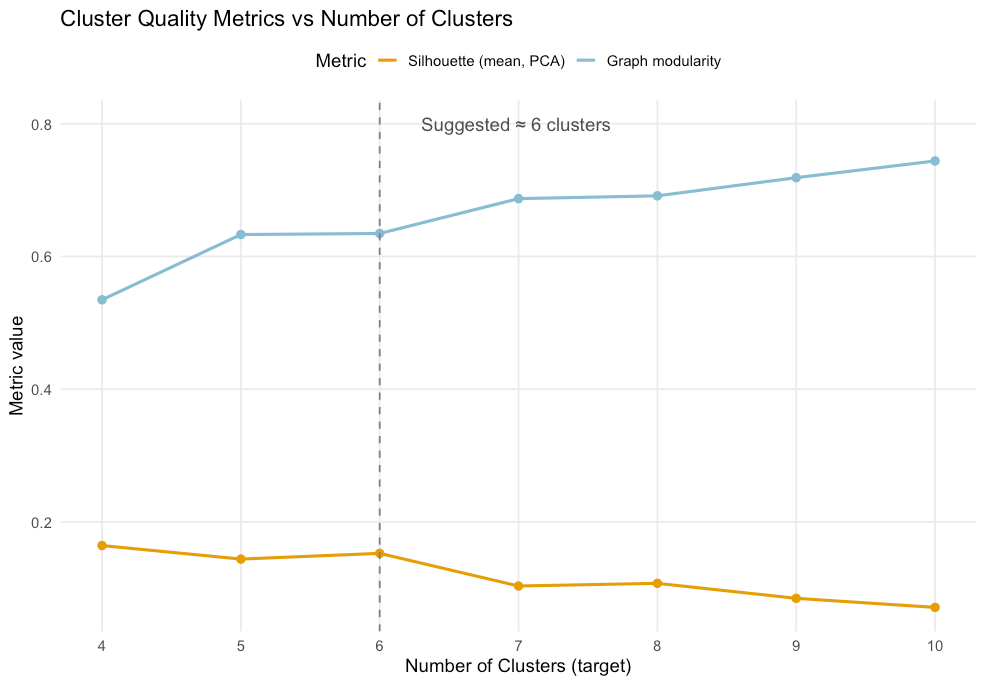


**Supplementary Figure 10 - Silhouette and Graph modularity scores.** To determine an appropriate clustering granularity, we generated cluster solutions across a range of resolution parameters resulting in 4 to 10 distinct clusters. We evaluated each one using two complementary metrics: (i) the mean silhouette score based on Euclidean distances in PCA space, and (ii) the graph modularity score of the shared nearest-neighbour (SNN) graph. The score was defined as: score = 0.6 × mean_silhouette + 0.4 × graph_modularity_score. Although this indicated that six clusters represented the statistically optimal partitioning, further examination of marker expression patterns and UMAP embeddings revealed that one cluster encompassed two biologically distinct subpopulations. To recover this structure, we selected the nearest resolution that produced seven clusters (resolution=0.23333).
